# Progress and Opportunities in the Characterization of Cellulose – An Important Regulator of Cell Wall Growth and Mechanics

**DOI:** 10.3389/fpls.2018.01894

**Published:** 2019-03-01

**Authors:** Sintu Rongpipi, Dan Ye, Enrique D. Gomez, Esther W. Gomez

**Affiliations:** ^1^Department of Chemical Engineering, The Pennsylvania State University, University Park, PA, United States; ^2^Department of Materials Science and Engineering, The Pennsylvania State University, University Park, PA, United States; ^3^Materials Research Institute, The Pennsylvania State University, University Park, PA, United States; ^4^Department of Biomedical Engineering, The Pennsylvania State University, University Park, PA, United States

**Keywords:** cellulose microfibrils, cellulose allomorphs, cellulose crystallinity, X-ray diffraction, X-ray scattering, vibrational spectroscopy, nuclear magnetic resonance spectroscopy, atomic force microscopy

## Abstract

The plant cell wall is a dynamic network of several biopolymers and structural proteins including cellulose, pectin, hemicellulose and lignin. Cellulose is one of the main load bearing components of this complex, heterogeneous structure, and in this way, is an important regulator of cell wall growth and mechanics. Glucan chains of cellulose aggregate via hydrogen bonds and van der Waals forces to form long thread-like crystalline structures called cellulose microfibrils. The shape, size, and crystallinity of these microfibrils are important structural parameters that influence mechanical properties of the cell wall and these parameters are likely important determinants of cell wall digestibility for biofuel conversion. Cellulose–cellulose and cellulose-matrix interactions also contribute to the regulation of the mechanics and growth of the cell wall. As a consequence, much emphasis has been placed on extracting valuable structural details about cell wall components from several techniques, either individually or in combination, including diffraction/scattering, microscopy, and spectroscopy. In this review, we describe efforts to characterize the organization of cellulose in plant cell walls. X-ray scattering reveals the size and orientation of microfibrils; diffraction reveals unit lattice parameters and crystallinity. The presence of different cell wall components, their physical and chemical states, and their alignment and orientation have been identified by Infrared, Raman, Nuclear Magnetic Resonance, and Sum Frequency Generation spectroscopy. Direct visualization of cell wall components, their network-like structure, and interactions between different components has also been made possible through a host of microscopic imaging techniques including scanning electron microscopy, transmission electron microscopy, and atomic force microscopy. This review highlights advantages and limitations of different analytical techniques for characterizing cellulose structure and its interaction with other wall polymers. We also delineate emerging opportunities for future developments of structural characterization tools and multi-modal analyses of cellulose and plant cell walls. Ultimately, elucidation of the structure of plant cell walls across multiple length scales will be imperative for establishing structure-property relationships to link cell wall structure to control of growth and mechanics.

## Introduction

The plant cell wall is a complex, heterogeneous network of several polymers and structural proteins. It provides mechanical strength and plays key roles in plant growth, cell differentiation, intercellular communication, water movement, and defense (Cosgrove, 2005). Most higher plants contain both primary and secondary cell walls. The primary cell wall is a thin, flexible, and highly hydrated structure that surrounds the growing cell, while secondary cell wall is a stronger and more rigid structure that starts to deposit when the cell ceases to grow. These cell wall types differ in function, rheological and mechanical properties, and in the arrangement, mobility, and structure of matrix polymers (Cosgrove and Jarvis, 2012). Primary walls are comprised of mainly cellulose, pectin, and xyloglucans with lesser amounts of arabinoxylans and structural proteins. Hydration of the pectin matrix facilitates the slippage and separation of cellulose microfibrils during expansive growth. The strength and rigidity of secondary walls come from a more oriented arrangement of cellulose microfibrils and the presence of lignin. Secondary cell walls are composed mainly of cellulose, lignin, xylans, and glucomannans, and are also less hydrated when compared to primary walls (Cosgrove and Jarvis, 2012).

Cellulose is the primary structural component responsible for much of the mechanical strength of the cell wall. The distribution and orientation of cellulose microfibrils within the cell wall contribute to the control of cell growth. The alignment of microfibrils provides the cell with mechanical anisotropy that enables preferential expansion in one direction (Jordan and Dumais, 2010). In addition to its biological significance, cellulose is an important raw material for textiles, paper, construction materials, and many industrially important chemical derivatives. It is also the most abundant carbohydrate on earth, and is a promising source for renewable energy.

The chemical structure of cellulose consists of linear chains of glucose units linked by β-1,4-glycosidic bonds. Glucan chains of cellulose aggregate via hydrogen bonds and van der Waals forces to form a long thread-like crystalline structure called a cellulose microfibril (Harris et al., 2010). Important structural properties of cellulose include crystallite shape and size and crystallinity. Many different analytical techniques have been employed to study the structure and assembly of cellulose microfibrils in cell walls, yet a comprehensive understanding over multiple length scales remains elusive.

**FIGURE 1:**
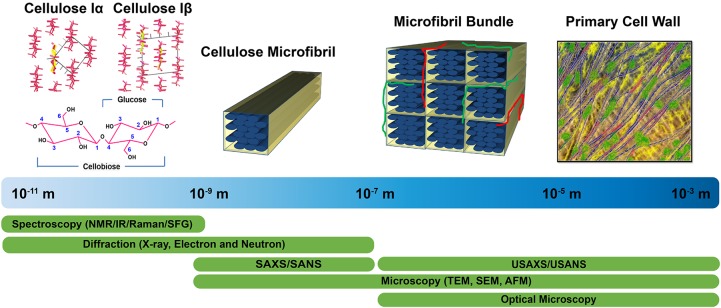
Tools enabling characterization of the primary cell wall at different length scales. Crystal structures of cellulose Iα and Iβ are reprinted with permission from Nishiyama et al. (2003). Crystal Structure and Hydrogen Bonding System in Cellulose Iα from Synchrotron X-ray and Neutron Fiber Diffraction. Journal of the American Chemical Society 125, 14300–14306. Copyright 2003 American Chemical Society. Primary cell wall is reprinted with permission from Cosgrove (2014). Re-constructing our models of cellulose and primary cell wall assembly. Current Opinion in Plant Biology 22, 122–131. Copyright © 2014 Elsevier Ltd. Schematic inspired by Martínez-Sanz et al. (2015a). Application of X-ray and neutron small angle scattering techniques to study the hierarchical structure of plant cell walls: A review. Carbohydrate Polymers 125, 120–134.

Structural characterization approaches currently used to examine plant cell walls are based on four broad categories of techniques: diffraction/scattering, spectroscopy, microscopy, and physicochemical assays. Figure 1 highlights these structural characterization tools and the length scales at which they can reveal information about cell wall structure. Solid state ^13^C nuclear magnetic resonance (NMR) studies led to the discovery of two cellulose allomorphs (VanderHart and Atalla, 1984). The crystal structures of cellulose Iα and Iβ were then determined with the help of X-ray, electron, and neutron diffraction studies (Sugiyama et al., 1991b; Abe et al., 1997; Nishiyama et al., 2002, 2003). Further details about structural differences between these two forms were described by Raman and Fourier-transform infrared (FTIR or IR) spectroscopy, which indicated that glucan chains have similar conformations but differ in hydrogen bonding patterns (Atalla and VanderHart, 1999). The selective detection of cellulose allomorphs is also possible through an emerging spectroscopic technique called sum frequency generation (SFG) spectroscopy (Kim et al., 2013). Beyond the crystal structure, X-ray diffraction (XRD), NMR, and IR and Raman spectroscopy are widely used to estimate the amount of crystalline cellulose present (degree of crystallinity) in plant cell walls. Crystallinity is also determined by some physico-chemical methods, such as the Updegraff method, iodine adsorption, sorption of water vapor, and enthalpy of wetting.

The supramolecular structure of the primary cell wall has been widely characterized by microscopic techniques. Many structural parameters such as crystallite size as well as fibril dimensions, cross-section, and spacing have been directly visualized (Cox and Juniper, 1973; Davies and Harris, 2003; Ding et al., 2014). Electron microscopy has been most widely used to image the fibrillar features of cellulose, but can nevertheless introduce artifacts during sample preparation. Therefore, other microscopic techniques, including scanning probe microscopy, fluorescence microscopy, confocal microscopy, and polarized light microscopy (Abe et al., 1997; Thomson et al., 2007; Choong et al., 2016), are now being explored to visualize the cell wall in its native state with minimal sample preparation.

Complementary to microscopy, the dimensions and packing of cellulose microfibrils are also examined by scattering and spectroscopic techniques (Fernandes et al., 2011; Newman et al., 2013; Zhang et al., 2016). Due to the minimal sample preparation required, scattering is ideal for characterizing the cell wall in its native state. Scattering approaches also offer the benefit of enabling investigation of a large size range, thus allowing for the arrangement of individual microfibrils as well as the aggregates of microfibrils to be examined.

Altogether, the combination of various techniques to characterize the organization of cell wall components opens the door to the examination of interactions between cellulose and other cell wall polysaccharides, potentially revealing various aspects of cell wall assembly (Martínez-Sanz et al., 2015a). For example, a combination of different imaging techniques such as atomic force microscopy (AFM), transmission electron microscopy (TEM), field emission scanning electron microscopy (FESEM), and confocal microscopy has been used to examine alteration in cellulose microfibril arrangement in the primary cell walls of the *Arabidopsis* xxt1 xxt2 double mutant that lacks detectable xyloglucan (Xiao et al., 2016). The study revealed that cellulose microfibrils are highly aligned in xyloglucan mutants as compared to those in wild type, suggesting that xyloglucan functions as a spacer between cellulose microfibrils in the primary cell wall.

This review summarizes techniques that are used for the characterization of structure and interactions of cellulose in plant cell walls, particularly cellulose crystallinity, microfibril size, and spatial organization along with cellulose–cellulose and cellulose-matrix interactions. We discuss both established and emerging techniques used for the molecular and microstructural characterization of cellulose structure, and highlight the strengths and limitations of each technique. In addition, the review introduces several characterization techniques that are presently not widely used for studying plant cell walls, but given their capabilities, might prove to be powerful tools to reveal new information regarding structure and organization.

## Crystalline Structure of Native Cellulose and Its Allomorphs

Six polymorphic forms of cellulose (Cellulose I, II, III_I_, III_II_, IV_I_, and IV_II_) that are interconvertible have been identified (O’Sullivan, 1997). Natural cellulose is found in the form of cellulose I, which has two allomorphs – cellulose Iα and cellulose Iβ (VanderHart and Atalla, 1984; Sugiyama et al., 1991a). Cellulose Iα is the dominant form in primitive organisms like bacteria and algae while Cellulose Iβ is dominant in higher plants. The existence of these two forms was established by spectroscopic techniques while their lattice structures were revealed by diffraction techniques. Both techniques are widely used to identify the two forms of cellulose in plant cell walls and they are also used to quantify the relative abundances of the cellulose forms. This section highlights studies that revealed the cellulose unit cell parameters by diffraction techniques, and also discusses methods for identifying the two different forms (cellulose Iα and Iβ) most commonly found in nature.

### Revealing the Unit Cell Parameters of Cellulose

The unit cell parameters of the two allomorphs of native cellulose were established through X-ray, electron, and neutron diffraction techniques. These techniques work on the principle of Bragg’s law to determine the *d*-spacing of atomic planes using electromagnetic waves. Thus, although diffraction data is often represented as intensity versus scattering angle θ, it is useful to represent it as a function of scattering vector *q* instead to normalize for the radiation wavelength λ (*q* = 4 π sin(*θ*/*2*)/λ). Diffraction techniques are used for two main purposes: (i) determination of the three-dimensional structure of molecules and thus their crystallographic form, and (ii) assessment of the degree of crystallinity. Due to the weak diffraction from primary cell walls, the majority of studies on the unit cell parameters have focused on cellulose from algae, bacteria, and secondary cell walls. We briefly discuss these findings in this section, but also emphasize available data on primary cell walls.

The first X-ray diffraction (XRD) patterns of cellulose fibers were collected from wood, hemp, and bamboo in 1913 (Nishikawa and Ono, 1913). The quantification of cellulose crystal parameters began with data derived from XRD of plant fibers including Ramie, hemp, flax, spruce, and cotton (Sponsler, 1928). The lattice parameters of cellulose from different sources like algae, bacteria, and plants are well summarized (O’Sullivan, 1997).

**FIGURE 2:**
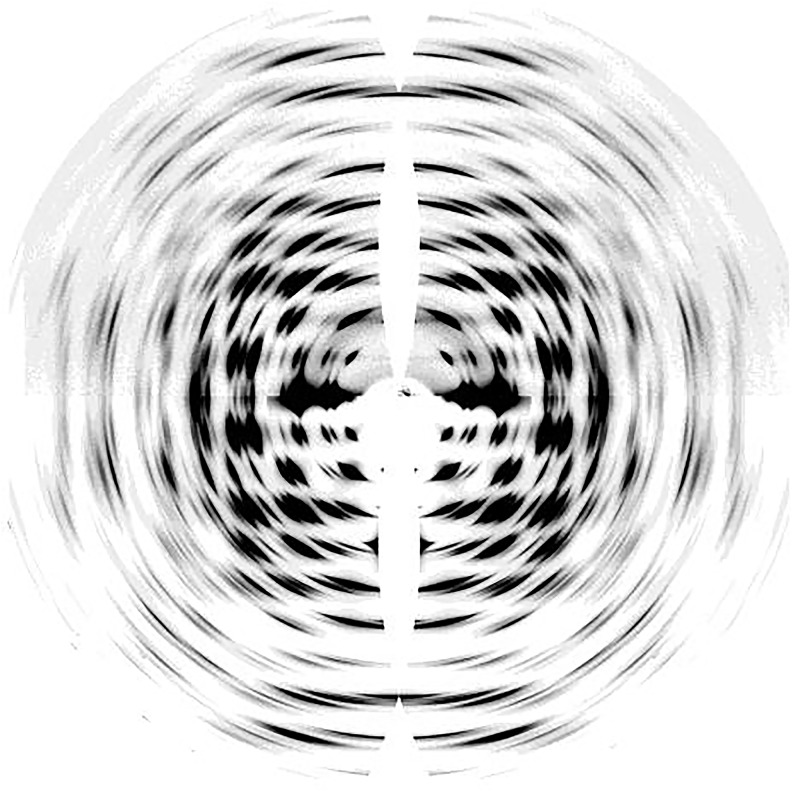
Synchrotron XRD 2D pattern of Halocynthia cellulose Iβ. Reprinted with permission from Nishiyama et al. (2002). Crystal Structure and Hydrogen-Bonding System in Cellulose Iβ from Synchrotron X-ray and Neutron Fiber Diffraction. Journal of the American Chemical Society 124, 9074–9082. Copyright © 2002, American Chemical Society.

Neutron diffraction (Beg et al., 1974; Ahmed et al., 1976) and electron diffraction (Honjo and Watanabe, 1958) studies have provided complementary structural information about cellulose I, enabling improvement of structural models developed from XRD data. Specifically, synchrotron X-ray techniques and neutron diffraction have enabled near atomic resolution. High-resolution synchrotron 2D data from oriented fibers of *Halocynthia*, which is nearly pure cellulose Iβ, is shown in Figure 2 (Nishiyama et al., 2002). The data have a resolution better than 1 Å with more than 300 unique reflections. The high resolution of this data was important to determine atomic coordinates in the unit cell of cellulose Iβ.

Synchrotron X-ray experiments can provide accurate locations for carbon and oxygen atoms, but cannot do so for hydrogen atoms due to their small X-ray scattering cross-sections. Neutron diffraction of intra-crystalline deuterated cellulose samples has revealed important information about the intermolecular hydrogen bond network in cellulose Iα and Iβ (Nishiyama et al., 2002, 2003). These experiments reveal that no inter-sheet hydrogen bonds exist in crystals of cellulose Iα and Iβ, and the sheets are held together by hydrophobic interactions and weak C-H⋅⋅⋅O bonds. The hydrogen bonds O3-H⋅⋅⋅O5 could be visualized through Fourier difference maps calculated from neutron diffraction data. These maps give information about missing atoms in the crystal structure by subtracting the calculated structure factors from observed ones. These studies also showed that within each cellulose sheet the intramolecular hydrogen bond at O3 is well organized while the intermolecular hydrogen bond for O2 and O6 is disordered over two possible networks. Furthermore, the relative occurrence of these networks differs in the two cellulose allomorphs. Also, the bond length and bond angle of the intrachain O3-H⋅⋅⋅O5 hydrogen bonds alternate between two different geometries in cellulose Iα and Iβ. While the alternating geometry of the bond is along the same chain in Iα, it is between two distinct chains in Iβ.

**FIGURE 3:**
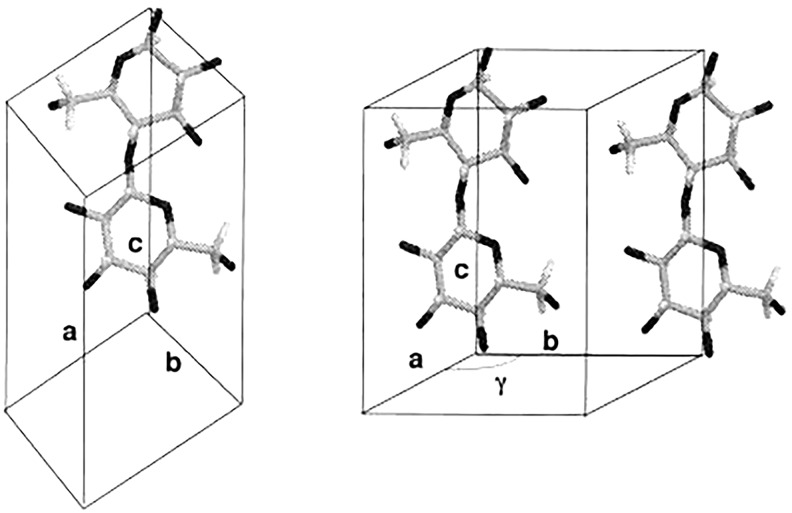
Chain packing in unit cell of cellulose Iα **(left)** and cellulose Iβ **(right)**. Reproduced from Koyama et al. (1997). Parallel-Up Structure Evidences the Molecular Directionality during Biosynthesis of Bacterial Cellulose PNAS 94 (17), 9091–9095. Copyright © 1997, The National Academy of Sciences of the United States.

Electron diffraction has made significant contributions in differentiating between the structures of the two crystalline phases of native cellulose, and established that cellulose Iα and Iβ have different lattice systems (Sugiyama et al., 1991a,b). Cellulose Iα has a triclinic lattice with one chain per unit cell and cellulose Iβ has a monoclinic lattice with two chains per unit cell, as shown in Figure 3. This technique has the advantage of producing intense diffraction patterns from a very small amount of sample, but the patterns can only be observed for a very short time for an organic substance like cellulose due to radiation damage caused by the electron beam.

High resolution synchrotron X-ray experiments have also been used to determine precise lattice parameters and the compositional ratio of cellulose Iα and Iβ in native cellulose from different sources including algae, bacteria, and plants (Wada et al., 1997). XRD peaks were deconvoluted using six types of profile functions such as Gaussian, Lorentzian, intermediate Lorentzian, modified Lorentzian, pseudo-Voigt, and Pearson VII. The pseudo-Voigt profile gave the best fit and was used to determine lattice spacings as shown in Table 1. The relative content of cellulose Iα was also determined based on the assumption that the first two equatorial reflections in the XRD pattern of *Valonia* cellulose are composites of cellulose Iα (100) and cellulose Iβ ($1\overset{¯}{1}0$), and of cellulose Iα (110) and cellulose Iβ (010) reflections. The two reflections were thus deconvoluted into four independent reflections using pseudo-voigt functions. The cellulose Iα content y_∝_ was then estimated as:

$$y_{\propto }=\frac{J_{I_{\alpha }100}+J_{I_{\alpha }010}}{J_{I_{\alpha }100}+J_{I_{\beta }1\overset{¯}{1}0}+J_{I_{\alpha }010}+J_{I_{\beta }110}}$$

**Table 1:** *d*-spacings of native cellulose calculated from synchrotron-based X-ray diffraction studies (Wada et al., 1997).

	*d*_1_, nm [composite of triclinic (100) and monoclinic ($1\overset{¯}{1}0$) reflections]	*d*_2_, nm [composite of triclinic (010) and monoclinic (110) reflections]	*d*_3_, nm [composite of triclinic (110) and monoclinic (200) reflections]
Valonia	0.610	0.531	0.392
Cladophora	0.611	0.531	0.392
Chaetomorpha	0.608	0.530	0.391
Bacterial cellulose	0.614	0.530	0.394
Halocynthia	0.601	0.535	0.390
Cotton	0.601	0.536	0.393
Ramie	0.597	0.534	0.394
Kouzo	0.596	0.534	0.393


where *J*_IiXXX_ denotes the integrated intensities *J* from Iα and Iβ reflections. The cellulose Iα fraction was found to be 0.65 for *Valonia* cellulose, which was nearly equal to the value of 0.64 reported for *Valonia* cellulose from ^13^C NMR (Yamamoto and Horn, 1994).

X-ray diffraction is perhaps more widely used to study cell walls than other techniques because of multiple reasons, including less sensitivity of the sample to radiation damage, easier sample preparation, and easier data acquisition when compared to electron diffraction, and the ability to examine samples without the need of deuteration when compared to neutron diffraction. Nevertheless, because large single crystals of cellulose are not readily available, XRD studies are typically performed using protocols for powder diffraction, and the final results can depend on the model assumptions. Also, one of the limitations of diffraction techniques is that their results are averaged over space and time. These techniques cannot provide a dynamic visualization of the cellulose structure that is required to explain some of its properties. The complementary use of various spectroscopy techniques, such as NMR, IR, Raman and, more recently, neutron spectroscopy, have been beneficial to elucidating cellulose structure. A recent report on inelastic neutron scattering of cellulose explored the dynamics of hydrogen bond networks (Araujo et al., 2018). The effects of increasing water content in kraft cellulose was observed in the inelastic neutron scattering bands that are assigned to the hydroxymethyl group. Formation of ice microcrystals due to shock-freezing led to partial disruption of the hydrogen-bond network, which could be concluded from shifts of the OH vibrational mode observed in the spectra.

### Identifying Allomorphs of Native Cellulose

**FIGURE 4:**
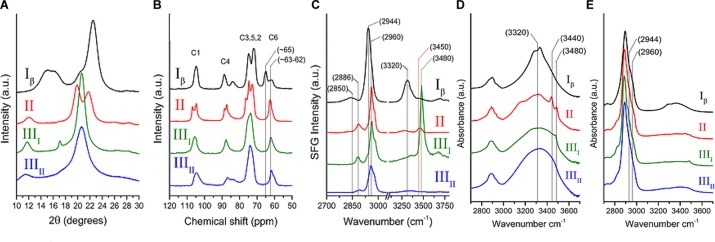
Techniques used to characterize cellulose polymorphism: **(A)** XRD, **(B)** NMR, **(C)** SFG, **(D)** IR, and **(E)** Raman. Reprinted by permission from RightsLink Permissions: Springer Nature. Cellulose. Cellulose polymorphism study with sum-frequency-generation (SFG) vibration spectroscopy: identification of exocyclic CH2OH conformation and chain orientation, Lee, C. M., Mittal, A., Barnette, A. L., Kafle, K., Park, Y. B., Shin, H., Johnson, D. K., Park, S., and Kim, S. H., Copyright © 2013.

The early crystallographic data of native cellulose from different sources were inconsistent with each other with respect to chain packing (French et al., 1987), and the assumption of twofold screw symmetry (P2_1_ space group) was inconsistent with reflections observed in electron diffraction (Atalla, 1987). Additionally, the findings from applying new spectroscopic techniques to cellulose could not be rationalized on the basis of the then existing crystallographic models. The inconsistencies were resolved through solid state (SS) ^13^C NMR spectral studies that led to the conclusion that native cellulose (cellulose I) is composed of two crystalline forms: cellulose Iα and Iβ (Atalla and Vanderhart, 1984). The two allomorphs are identified in plant cell walls, through spectroscopic and diffraction techniques as discussed in the following section. Figure 4 shows the XRD pattern and spectra obtained from NMR, SFG, IR, and Raman spectroscopy for different forms of cellulose. These techniques present spectra with distinct features for each of the allomorphs and can be used to estimate the relative contents of the forms of cellulose in a sample.

NMR spectroscopy provides qualitative and quantitative information about atoms in a sample and their chemical environments. The technique can distinguish between chemically equivalent carbons located at magnetically non-equivalent sites. The application of Cross-Polarization Magic Angle Spinning (CP/MAS) ^13^C NMR to study cellulose revealed that cellulose Iα and Iβ can be differentiated in the NMR spectra based on the multiplicity of the C4 resonance peak near 88–90 ppm. Cellulose Iα has a second peak in the down-field region while cellulose Iβ has it in the up-field region. The relative abundance of the allomorphs is calculated by deconvolution of the resonance peaks in the C4 region (Yamamoto and Horii, 1993). Figure 4B shows the NMR spectra of cellulose Iβ in comparison to other forms of cellulose. Cellulose I, II, and III can be distinguished on the basis of the chemical shifts of the C6 resonance peak; they have signals at 65.5–66.2, 63.5–64.1, and 62.1–62.8 ppm, respectively (Isogai et al., 1989).

IR and Raman spectroscopy are vibrational spectroscopic techniques that can provide complementary information on chemical functionality, molecular conformation, and hydrogen bonding. IR spectroscopy requires a dipole change while Raman requires a polarizability change as a molecule rotates or vibrates. One key advantage of Raman over IR spectroscopy for the study of hydrated cell walls is that water appears as broad absorption bands in IR spectra, while water bands have weak intensities in Raman spectra. Moreover, changes in the refractive index of the material can cause variations in IR background but not in Raman, because excitation frequencies are far from absorption bands (Agarwal, 2014). When comparing the IR and Raman spectra of cellulose Iα and Iβ, differences are observed in the OH-stretching region (3200–3600 cm^-1^). In IR spectra, cellulose Iα has peaks at 3240 and 750 cm^-1^ while cellulose Iβ has peaks at 3270 and 710 cm^-1^ (Sugiyama et al., 1991a). These findings suggest that the two phases have similar chain conformations, but differ in hydrogen bonding patterns and dihedral angles at the glycosidic linkages. Line shape analyses of these characteristic peaks can be carried out to determine the mass fractions of cellulose Iα and Iβ in various cellulose samples (Yamamoto et al., 1996). Figure 4D,E compare the IR and Raman spectra of cellulose Iβ with the spectra obtained for cellulose II, III_I_, and III_II_. The main differences in the spectra are seen for the region above 3000 cm^-1^. Cellulose Iβ has a distinct peak at about 3320 cm^-1^, cellulose II has two peaks at about 3450 and 3480 cm^-1^, cellulose III_I_ has one peak at about 3480 cm^-1^_,_ while cellulose III_II_ has no distinct sharp peak in this region.

Sum frequency generation (SFG) vibrational spectroscopy is a non-linear optical spectroscopy tool that is sensitive to non-centrosymmetric crystalline materials. As discussed in the Crystallinity of Cellulose, Spectroscopic Techniques Section, SFG is sensitive to structural ordering over an optical coherence length that enables it to characterize the structural hierarchy of cellulose microfibrils in the cell wall (Kim et al., 2013). NMR, IR, and Raman spectroscopy are widely used to study the conformation of purified cellulose, but their application is limited when it comes to native cellulose or lignocellulosic biomass, where spectral interference from other cell wall components cannot be avoided. The non-centrosymmetric requirement of SFG negates the interferences from SFG-inactive groups and thus enables the identification of exocyclic CH_2_OH conformation and chain orientation of forms of cellulose as shown in Figure 4C (Lee et al., 2013). Similar to IR and Raman spectroscopy, SFG also exhibits characteristic peaks for cellulose Iα at 3240 cm^-1^ and for cellulose Iβ at 3270 cm^-1^ (Lee et al., 2015b).

## Crystallinity of Cellulose

Crystallinity is the ratio of crystalline to crystalline plus amorphous content by volume, and as such is a measure of structural order. Crystallinity affects mechanical properties such as strength and stiffness of cellulose and cellulose-derived materials. Higher cellulose crystallinity results in increased Young’s modulus, tensile strength, density, and hardness (Lionetto et al., 2012). It is also an important parameter in many micromechanical models for wood (Bergander and Salmén, 2002; Hofstetter et al., 2005). Furthermore, the relative level of crystalline versus amorphous material within cellulose can influence the accessibility and reactivity of a given cellulose substrate to enzymes for biomass conversion. Given the importance of this metric, the crystallinity of cellulose has been estimated by many techniques, including XRD, IR and Raman spectroscopy, SS-NMR, SFG spectroscopy, Differential Scanning Calorimetry (DSC), and a variety of physicochemical assays. The measured crystallinity of cellulose can vary significantly depending on the technique and analysis approach used, with variations of up to 30–40% in reported values for cellulose-based materials (Thygesen et al., 2005; Park et al., 2010; Kljun et al., 2011; Agarwal et al., 2013; Karimi and Taherzadeh, 2016). Table 2 summarizes the crystallinity of cellulose derived from different sources as determined by XRD and NMR (Park et al., 2010). The lack of consensus reflects the challenges in measuring the degree of order in plant cell walls and the limitations of the aforementioned techniques, which we discuss below.

**Table 2:** Crystallinities of cellulose from different sources determined by XRD and NMR analysis methods.

Cellulose sample	XRD^∗^			NMR^∗∗^
		
	Peak height	Peak deconvolution	Amorphous subtraction	C4 peak separation
Bacterial microcrystalline cellulose	95.2	73.1	82.4	73.8
Avicel PH-101	91.7	60.6	77.7	56.7
SigmaCell 50	91.2	61.3	79.4	56.1
SigmaCell 20	84.8	64.2	67.0	52.6
JT Baker cellulose	85.5	61.5	69.1	49.1
Fluka cellulose	82.9	52.9	61.6	48.6
SolkaFloc cellulose	78.3	56.8	57.2	43.9
Sigma α-cellulose	78.0	55.9	54.4	41.5


**^∗^XRD: X-ray Diffraction; ^∗∗^NMR: Nuclear Magnetic Resonance Spectroscopy. All values are means (Park et al., 2010).**

### Physicochemical Methods

The Updegraff method is a commonly used chemical method for determining the amount of crystalline cellulose in a sample (Updegraff, 1969). This method involves extraction of lignin, hemicellulose, and xylosans with an acetic acid/nitric acid reagent, leaving behind crystalline cellulose. Cellulose is then dissolved in 67% H_2_SO_4_, and the amount of crystalline cellulose can be determined after treatment with an anthrone reagent to enable colorimetric analysis (Scott and Melvin, 1953; Kumar and Turner, 2015).

In principle, cellulose crystallinity should be related to accessibility. The moisture sorption of cellulose takes place primarily by hydrogen bonding of water to accessible hydroxyls in less ordered regions at the surfaces of elementary fibrils and their random fibrillar aggregations at relative humidities lower than 50–60%. Thus, moisture regain of cellulose is a more direct measure of cellulose accessibility to reactants, rather than crystallinity. It is common practice to relate accessibility to crystallinity through the following equation (Howsmon, 1949):

A=σX+(100−X)

where *A* is the percentage of accessible cellulose in the sample, *σ* is the fraction of accessible cellulose on the surface of crystalline regions, and *X* is the percentage of crystalline cellulose in the sample.

The determination of accessibility of glucan chains based on deuterium exchange is based on the assumption that accessible OH groups in amorphous regions of cellulose readily exchange their hydrogen atoms for deuterium while the OH groups in crystalline regions exchange more slowly. Accordingly, the reaction curve for exchange reactions has two separate regions: an initial rapid rate region followed by a slow rate regime (Frilette et al., 1948), and the crystallinity has been related to accessibility similarly as shown in equation 2.

Because iodine is reported to be adsorbed in the amorphous regions of cellulose, measurements of iodine adsorption have also been used to determine crystallinity (Hessler and Power, 1954). The amount of iodine adsorption per gram of cellulose has been linked to the fraction of amorphous cellulose within a sample. The crystallinity was then estimated by subtracting the amorphous fraction from 100.

A recent report has attempted to calculate the absolute degree of crystallinity of cellulose based on sorption of water vapor and enthalpy of wetting (Ioelovich, 2016). The crystallinity *x* of cellulose is calculated from sorption of water using the following equation that is derived from the sigmoidal isotherm of sorption of water vapor by semi-crystalline cellulose:

x=1−2A(1−2.61 In φ)

where *A* is the relative amount of water in cellulose by mass and φ is the relative vapor pressure at a constant temperature of 25°C. Under the assumption that water molecules interact with amorphous domains of cellulose and this interaction is accompanied by release of heat, the enthalpy of wetting is directly proportional to the amount of amorphous cellulose content. Then the crystallinity can also be determined by:

$$x=1-\frac{\Delta H}{\Delta H_{am}}$$

where Δ*H*_am_ is the enthalpy of wetting of purely amorphous cellulose. A value of Δ*H*_am_ = -167.5 J/g has been reported and used to estimate crystallinity (Ioelovich, 2016). The crystallinity of microcrystalline cellulose samples was found to range from 0.72 to 0.75, as determined from the enthalpy of wetting and water sorption methods.

When compared to the crystallinity found from XRD measurements, physicochemical methods typically report a higher value of crystallinity. One possible origin of the discrepancy is the compositional and structural heterogeneity of cell walls, in particular of primary cell walls, that might complicate access to non-crystalline components. This would invalidate the assumption of a direct relationship between crystallinity and the physical and chemical properties investigated by these methods.

### X-Ray Diffraction

**FIGURE 5:**
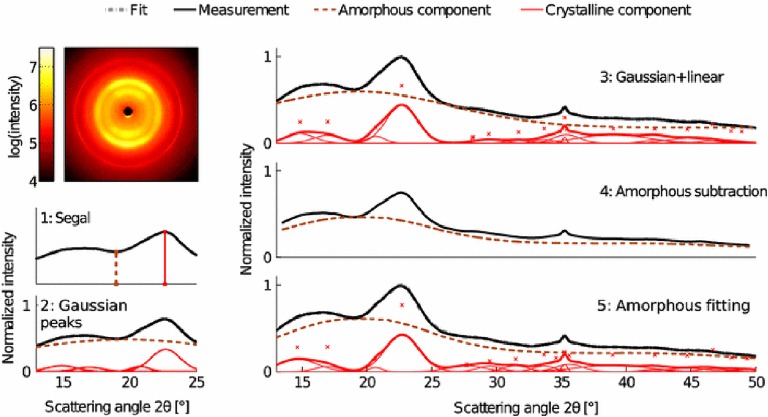
X-ray diffraction methods for determination of the crystallinity of cellulose. Reprinted by permission from RightsLink Permissions: Springer Nature. Cellulose. Comparison of sample crystallinity determination methods by X-ray diffraction for challenging cellulose I materials, Ahvenainen, P., Kontro, I., and Svedström, K., Copyright © 2016.

X-ray diffraction is the most widely used technique for determining the crystallinity of cellulose due to its established reliability and accuracy, and minimal sample preparation requirements. XRD gives a measure of crystallinity as the mass fraction of crystalline cellulose within the entire sample (Ahvenainen et al., 2016). As shown in Figure 5, three methods are widely used for estimation of crystallinity from XRD, including: (i) the peak height or Segal method; (ii) peak deconvolution of crystalline and amorphous peaks; and (iii) the amorphous subtraction or Ruland–Vonk method. These approaches are discussed extensively in various reviews (Park et al., 2010; Kim et al., 2013; Ju et al., 2015; Karimi and Taherzadeh, 2016) and are described briefly below.

The peak height method, also called the Segal method (Segal et al., 1959), is the most widely used analysis approach to characterize the crystallinity of cellulosic samples. The crystallinity *x* is calculated by:

$$x=\frac{I_{200}-I_{AM}}{I_{200}}$$

where *I*_200_ is the height of the (200) peak and *I*_AM_ is the height of the minimum between the (200) and (110) peaks. This method is not very accurate as the exact amount of the crystalline fraction is proportional to the peak area rather than to the peak height. Also, the underlying assumption of equation 5 is that scattering intensities from amorphous and crystalline content are equivalent per unit volume, which actually depends on the details of the structure factor of each of these phases. As a consequence, the crystallinity obtained using this method is dependent on crystallite size and cellulose allomorph (Ju et al., 2015).

The second method is based on peak deconvolution of crystalline and amorphous peaks. In XRD data, crystalline cellulose is represented by several intense peaks at (110), (102), (200), and (004) for cellulose Iβ and a single broad peak for the amorphous phase. Gaussian, Lorentzian, and Voigt functions are commonly used for peak fitting and the ratio of the area of the crystalline peaks to the total area is defined as the crystallinity. The accuracy of this method depends on selecting the correct peaks that correspond to the actual diffraction contributed by each fraction.

In the third method, also called the amorphous subtraction or Ruland–Vonk method (Ruland, 1961), the crystallinity is defined as the ratio of an area above an amorphous profile to the total area. The amorphous profile is obtained either from a polynomial function or a pattern measured from experimentally prepared material believed to be entirely amorphous, such as ball-milled cellulose, regenerated cellulose, xylan, or lignin powder. In this method, a scaling factor is applied to the amorphous spectrum so that after subtraction from the original spectrum, no negative signal occurs in the residual spectrum. Often, the scaled amorphous background touches the diffractogram somewhere in the low *q* (low *2*θ) region where the intensity is most poorly determined due to the fine adjustment of slits and the effects of axial divergence, so the method is sensitive to instrumental inaccuracies. It can also be difficult to compare samples of different origin. In addition, it can be challenging to compare results from different studies due to the variability in the amorphous standard used.

The crystallinity obtained from XRD can depend on crystallite size and preferred orientation of crystallites. The use of area-based fitting methods can better avoid the effects of crystallite size than peak height-based methods. The effects of preferred orientations can be mitigated by use of 2D Rietveld refinement, which includes the contribution of all diffraction peaks and two-dimensional diffraction data. Both 1D and 2D Rietveld refinement of XRD data are reported to accurately determine the degree of crystallinity (Thygesen et al., 2005; De Figueiredo and Ferreira, 2014; Driemeier, 2014). Because 2D Rietveld analysis is done on 2D diffraction data, it takes into account the preferred orientation and thus is considered more accurate for textured samples (Ahvenainen et al., 2016).

Additional approaches to estimate the crystallinity of cellulose from XRD data have also been developed, including the Hermans–Weidinger method (Gusev, 1978) and the Debye method (Thygesen et al., 2005), although these approaches are less widely used in comparison to the abovementioned analyses. The Hermans–Weidinger method was developed for the determination of polymer crystallinity based on the proportionality of X-ray scattering intensities of crystalline and amorphous parts of a polymer. The proportionality is expressed as:

$$\frac{x_{1}}{x_{2}}=\frac{I_{c_{1}}}{I_{c_{2}}}\,\left(6\right)$$

where *x*_I_ is the degree of crystallinity and *I*_cI_ is the scattering intensity from the crystalline region. Crystallinity of a sample (labeled 1 in equation 6) can be determined only when a sample of known crystallinity (labeled 2) is available. The Debye method is similar to the Rietveld refinement method with the difference being that it requires simulation and fitting of the diffractogram to the experimental data to determine the quality of the fit (Thygesen et al., 2005). This approach has an advantage over the Rietveld method as the crystallite dimensions are included explicitly in the simulations and not fitted by analytical peak profile functions. This enables the Debye method to give the most reliable estimate of the crystalline part of the diffraction pattern, but it is less commonly used due to the heavy computing efforts required.

A robust estimate of the crystallinity from XRD measurements requires consideration of various approaches for data analysis. Even then, the limitations highlighted above preclude confidence in absolute values, although relative values for the crystallinity can reveal trends in samples that differ minimally (e.g., within the same species). Often, the term “crystallinity index” is used for crystallinities obtained from XRD to emphasize the challenges with comparing these values to those extracted from other techniques.

### Spectroscopic Techniques

The intra- and inter-molecular hydrogen bonds found in crystalline cellulose can be analyzed using IR spectroscopy. The absorption band between 1420 and 1430 cm^-1^ (*A*_1430_) is assigned to a symmetric CH_2_ bending vibration, known as the “crystallinity band,” and the band appearing between 893 and 898 cm^-1^ (*A*_898_) is assigned to C–O–C stretching at β-(1→4)-glycosidic linkages, known as the “amorphous band” (Nelson and O’Connor, 1964). Two terms related to crystallinity of cellulose have been defined, namely Lateral Order Index (LOI) and Total Crystallinity Index (TCI). LOI, also called the empirical crystallinity index, is the ratio of the intensities of *A*_1430_ to *A*_898_ and is sensitive to the amount of crystalline versus amorphous regions in cellulose. A lower LOI indicates a more amorphous structure (O’Connor et al., 1958). TCI is the ratio of the absorption band at 1372 to 2900 (Nelson and O’Connor, 1964; Poletto et al., 2014). The band at 1372 cm^-1^ is assigned to C-H bending and is reported to be affected by the amorphous content of a cellulose sample while the band at 2900 cm^-1^ is assigned to C-H and CH_2_ stretching and is reported to be unaffected by changes in crystallinity. Taking the ratio of intensities of these bands as TCI enables the crystallinity index to be insensitive to sources of variation other than changes in crystallinity. IR spectroscopy is routinely used to characterize woody biomass meant for biofuel conversion (Amiri and Karimi, 2015; Noori and Karimi, 2016).

Different peak ratios in Raman spectra have been reported in literature as a measure of crystallinity. The relative intensity ratios of the Raman bands 1481 and 1462 cm^-1^ in cellulose I (Schenzel et al., 2005) and that of 380 and 1096 cm^-1^ bands (Agarwal et al., 2010) are both reported as measures of the crystallinity. Unfortunately, both IR and Raman spectroscopy face challenges when characterizing the crystallinity present in primary cell walls due to the interference of signals from other wall components.

In the ^13^C NMR spectra of cellulose, the peak at 89 ppm is assigned to C4 in crystalline cellulose and the peak at 84 ppm to amorphous cellulose (Atalla and VanderHart, 1999). The crystallinity from NMR spectra is defined as the integral area of the C4 peak from 87 to 93 ppm divided by the total integral area assigned to the C4 peaks (from 80 to 93 ppm). This method has been used to determine the degree of crystallinity in wood (Newman and Hemmingson, 1990; Newman et al., 1993) and to study the effect of crystallinity on enzymatic degradation of cellulose (Mansfield and Meder, 2003). It has also been applied to estimate crystallinity in primary cell walls of cellulose synthase mutants of *Arabidopsis thaliana* (Harris et al., 2012).

As introduced earlier, the non-centrosymmetric requirement of SFG allows selective detection of cellulose in plant cell walls and characterization of its structural properties. SFG has also been used to determine the amount of crystalline cellulose in secondary cell wall samples, which was estimated by applying a calibration curve from Avicel to the intensity of the CH_2_ SFG peak of cellulose at 2945 cm^-1^ (Barnette et al., 2012). The limitations of this technique lie in the assumption of 100% crystalline Avicel, the assumption of the same signals from Avicel cellulose and from the biological systems under study, and the neglect of the effect of crystal size. Perhaps as a consequence, the technique has not yet been reported for crystallinity studies of primary cell walls.

## Cellulose Microfibril Size and Organization

Direct visualization of the cell wall through light microscopy shows the existence of cellulose in a bundled fibrillar structure. High resolution electron microscopy reveals microfibrils that are aggregated, such that individual microfibrils (sometimes termed elementary fibrils) have cross-sections of 2–4 nm and lengths of 100 nm or more (Kraissig, 1992). Complete understanding of this fibrillar network requires the characterization of structural parameters, including fibril length, lateral size and shape, as well as the spatial arrangement of microfibrils. These parameters have a strong influence on the mechanical and physicochemical properties of cellulose and its derivatives. The following section discusses the characterization of the abovementioned parameters through different techniques such as microscopy, diffraction/scattering, spectroscopy, and chemical methods. We cover examples from studies of bacterial cellulose, primary cell walls, and secondary cell walls.

### Size and Shape of Cellulose Microfibrils

Perhaps the simplest approach to estimate the dimensions of microfibrils relies on physicochemical methods. Under the assumption that the microfibril length is equal to the chain length, the length is estimated from the degree of polymerization (DP) of residual cellulose that remains after an initial drastic drop upon dissolution in dilute acid. This degree of polymerization is called the leveling off DP, and the crystallite length is estimated as the product of the leveling off DP and length of one monomer unit. The DP of cellulose has also been determined through light scattering, osmotic pressure, and gel permeation chromatography (Levi and Sellen, 1967; Holt et al., 1973). The crystallite width is calculated by observing the reactivity of cellulose toward dilute mineral acid and deuterium oxide. Under the hypothesis that both acid hydrolysis and deuteration take place in the amorphous regions, but only deuteration takes place on the surface, the number of molecules per side of a rectangular cross-section is calculated and multiplied by the average of the (101) and ($10\overset{¯}{1}$) spacings for cellulose I. For example, values for the crystallite width are 31 Å for cotton and 33 Å for Ramie, with crystallite lengths of about 100 nm for both (Scallan, 1971). As discussed below, these crystallite widths are consistent with measurements from electron microscopy and other techniques.

Various approaches have attempted to directly image the size and shape of microfibrils (Table 3). The use of electron microscopy along with techniques like metal shadowing (Bayley et al., 1957; Beer and Setterfield, 1958), negative staining (Heyn, 1966; Revol, 1982; Manley, 2003), and diffraction contrast imaging (Bourret et al., 1972; Revol, 1982) have revealed valuable structural information about cellulose from several sources including valonia, jute, cotton, and ramie fibers. Based on the findings from X-ray diffraction/scattering and electron microscopy of cellulose materials following different chemical treatments, two descriptions of microfibrils developed. One hypothesis stated that each microfibril has a single crystalline core whose size is almost the same as a microfibril, while an alternative hypothesis stated that each microfibril was composed of elementary microfibrils of 35 Å width (Nieduszynski and Preston, 1970). The former hypothesis was supported with studies on bacterial cellulose, where apparent crystallite lateral dimensions are much larger than 35 Å, and not necessarily in its multiples. Cellulose crystallites from *Chaetomorpha melagonium* and *Acetobacter xylinum* were found to measure between 100 and 200 Å when studied through X-ray diffraction and electron microscopy (Colvin, 1963; Nieduszynski and Preston, 1970). Further work based on high resolution imaging techniques was crucial to resolve these conflicting descriptions of cellulose organization, as described below.

**Table 3:** Microfibril diameter from different sources of cellulose obtained through the use of different analytical characterization techniques.

Source of cellulose	Microfibril diameter (nm)	Techniques^∗^
*Arabidopsis thaliana*	5.8 ± 0.17	AFM (Davies and Harris, 2003)
Celery collenchyma	2.4–3.6	NMR, SAXS, WAXS (Kennedy et al., 2007a)
	2.9–3.0	SANS, WAXS (Thomas et al., 2013)
	2.6–3.0	SAXS (Kennedy et al., 2007b)
Celery parenchyma	6.0–25.0	AFM (Thimm et al., 2000)
Cotton	2.5-4.0	TEM (Heyn, 1966)
	4.9–6.1	TEM (Nieduszynski and Preston, 1970)
	5.5	SAXS (Heyn, 1955)
Flax fiberes	1.0–5.0	SAXS (Astley and Donald, 2001)
	2.8	SAXS (Heyn, 1955)
Jute	2.8	TEM (Heyn, 1966)
	2.8	SAXS (Heyn, 1955)
Maize	3.2 – 5.3	AFM (Ding and Himmel, 2006)
	2.5 – 3.5	WAXS, NMR (Rondeau-Mouro et al., 2003)
Mung bean	2.5 – 3.2	WAXS, NMR (Newman et al., 2013)
Oak wood	2.9 – 3.1	WAXS, SAXS (Svedström et al., 2012)
Onion	8.0 – 10.0	NMR (Ha et al., 1998)
	4.4 ± 0.13	AFM (Davies and Harris, 2003)
Quince	2.0	NMR (Ha et al., 1998)
Ramie	3.6 – 4.8	TEM (Heyn, 1966)
	5.9	TEM (Nieduszynski and Preston, 1970)
	4.3	SAXS (Heyn, 1955)
Spruce wood	2.5	TEM, WAXS, SAXS (Jakob et al., 1995)
	2.9	WAXS (Andersson et al., 2000)
	3.1 – 3.2	SANS, WAXS (Fernandes et al., 2011)
	2.9 – 3.1	WAXS (Peura et al., 2007)
Sugi wood	2.4 – 2.6	SAXS (Suzuki and Kamiyama, 2004)
Tunicin	3.4 – 7.6	TEM (Nieduszynski and Preston, 1970)
*Valonia ventricosa*	18.0	TEM (Revol, 1982)
	10.0 – 20.0	WAXS (Caulfield, 1971)
	3.0	WAXS, NMR, IR (Horikawa et al., 2009)


**^∗^AFM, atomic force microscopy; NMR, nuclear magnetic resonance spectroscopy; SAXS, small angle X-ray scattering; SANS, small angle neutron scattering; WAXS, wide angle X-ray scattering (synonymous with XRD); TEM, transmission electron microscopy, IR, infrared spectroscopy*.*

**FIGURE 6:**
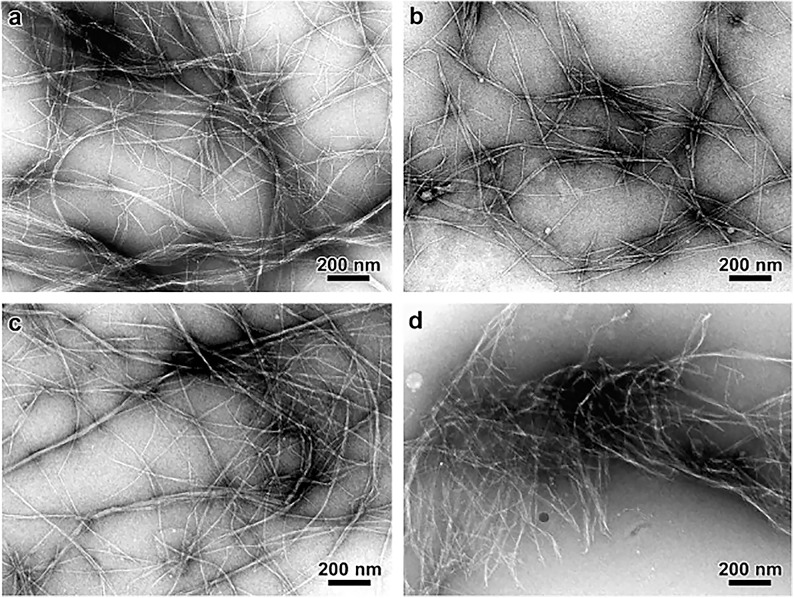
Transmission Electron Microscopy micrographs comparing the morphology of cellulose microfibrils isolated by different chemical treatments. **(a)** Peroxide alkaline, **(b)** peroxide-alkaline-hydrochloric acid, **(c)** 5 wt% potassium hydroxide, and **(d)** 18 wt% potassium hydroxide. The combination of peroxide alkaline and hydrochloric acid or the application of a high concentration (18 wt%) potassium hydroxide solution leads to shorter microfibrils, suggesting these treatments can cause microfibril scission. Reprinted from Carbohydrate Polymers, 76, Zuluaga, R., Putaux, J. L., Cruz, J., Vélez, J., Mondragon, I., Gañán, P. Cellulose microfibrils from banana rachis: Effect of alkaline treatments on structural and morphological features, 51–59, Copyright © 2009, with permission from Elsevier.

Lattice imaging of native cellulose from ramie fibers and different algal and bacterial sources was made possible with high resolution electron microscopy in combination with negative staining, metal shadowing, and diffraction contrast imaging (Sugiyama et al., 1985; Kuga and Brown, 1987a,b). These studies established that each microfibril corresponds to a single crystalline entity. Negative staining of sections of cellulose from cotton, ramie, and jute fibers revealed lateral dimensions between 25 and 40 Å (Heyn, 1966). As shown in Figure 6, transmission electron microscopy (TEM) with negative staining has also been used to demonstrate individual cellulose microfibrils that result from various alkaline treatments of vascular bundles of banana rachis (Zuluaga et al., 2009). Using electron diffraction and dark field electron microscopy, cellulose crystallites from algae (*Valonia ventricosa*) were found to be above 1000 Å in length and 140 to 180 Å in width (Bourret et al., 1972). Thus, although the “elementary” unit appears to be a microfibril of a few nanometers, dimensions of cellulose crystallites appear to vary depending on the source. In a similar way, no agreement has been reached on the cross-sectional shape of cellulose found from imaging. The cross-section of valonia microfibrils was found to be almost square-shaped with an average size of 180–200 Å (Revol, 1982; Sugiyama et al., 1985) while the cross-section of tunicate cellulose was found to be parallelogram shaped (Helbert et al., 1998a,b). Even though valuable information has been obtained about cellulose microfibrils from electron microscopy, the sample preparation that generally requires drying could introduce artifacts through modifications in the physical structure of native cellulose, such as collapse and aggregation of microfibrils. This has limited the study of microfibril shape and diameter in primary cell walls through TEM.

As an alternative to electron microscopy, scanning force microscopy (SFM), also termed atomic force microscopy (AFM), and optical microscopy are techniques that can visualize cellulose microfibrils with spatial resolution ranging from the micrometer to the sub-nanometer scale in biologically relevant environments. AFM techniques reveal the surface topology by measuring the interaction between a fine physical probe and the surface of the sample. Imaging contrast is based on variations of the sample topology, modulus, or interaction with the probe. AFM can record the surface topography and properties at the nanoscale by scanning a sample under a sharp stylus or tip, which is often made from silicon or silicon nitride. The stylus is attached to a cantilever, which is deflected as the stylus interacts with the surface. Images are produced by measuring the deflection of the cantilever as the sample is scanned. Alternatively, atomic force microscopes can be operated in constant-force mode in which a feedback system is used to keep the deflection constant (Prater et al., 1990). AFM enables direct characterization of sample surfaces with high spatial resolution (0.1–100 nm) and minimal sample preparation; thus, AFM is ideal for characterizing the structure of cell walls, as many features can be detected within this resolution range (Yarbrough et al., 2009). Samples need not be fixed, stained, dried, or metal coated as in the case of electron microscopy. Even if a pectin layer is present, the tip can probe through this soft layer to reveal the microfibril structure underneath in primary cell walls (Zhang et al., 2014, 2016).

The earliest cellulose-containing biological samples studied using AFM were dried cells of archae-bacterium *Halobacterium halobium* (Butt et al., 1990); later studies focused on bacterial polysaccharides (Gunning et al., 1995) and cellulose from root hair cell wall of *Zea mays* and *Raphanus sativus* (van der Wel et al., 1996). AFM has also been used to visualize cellulose microfibrils in hydrated primary cell walls from apple, water chestnut, potato, and carrot (Kirby et al., 1996). These measurements supported the polylaminate description of cell wall structure. Furthermore, the effect of hydration on the diameter of cellulose microfibrils in celery parenchymal cell walls was studied using AFM. It was found that the measured diameters depend on the water content of the samples and also on the procedure of dehydration, with diameters ranging from 15.2 ± 0.4 nm before dehydration to 25.1 ± 0.8 nm after dehydration (Thimm et al., 2000). Nevertheless, as the tip scans across the surface, it can lead to broadening of lateral features due to the width of the tip itself, leading to differences in measured microfibril diameters from AFM in comparison to other techniques. Measuring the height of microfibrils (in the z-direction) resolves this problem, as was done to find the dimensions of cellulose microfibrils from partially hydrated cell walls of onions and *A. thaliana* (Davies and Harris, 2003). Microfibrils were 4–6 nm in diameter and contain a single cellulose crystallite, 2–3 nm wide, which is surrounded by non-cellulosic polysaccharides. It was also found that removal of pectin from the cell wall improved the accuracy of measurements. AFM studies of maize parenchyma cell wall indicated microfibril dimensions similar to that found in onion and *A. thaliana* as discussed above, although the authors proposed a 36-chain model for each microfibril (Ding and Himmel, 2006). AFM has also been used to compare cellulose microfibrils in different scales of onion (Kafle et al., 2014; Tittmann and Xi, 2014; Zhang et al., 2014). These studies showed that the microfibrils are more ordered in older scales than in younger scales. Altogether, previous work has demonstrated AFM as a powerful tool for imaging of the cell wall in physiological environments.

Scanning Electron Microscopy (SEM) is an alternative approach to image the surface of plant cell walls. Sample preparation for SEM is simpler than for TEM, because electron-transparent samples and heavy metal staining are not required. SEM allows imaging cell walls directly and has been used to observe the cell wall structure of both primary (Crow and Murphy, 2000; Carpita et al., 2001) and secondary cell walls (Awano et al., 2002; Kim et al., 2012). Measurements of microfibril dimensions are consistent with estimates derived from AFM (Zheng et al., 2018). Nevertheless, SEM usually requires dehydration or critical-point drying, removal of the top pectin layer (if present), as well as deposition of a conductive coating to prevent charging, which may cause artifacts. As a consequence, the technique is often used to complement other microscopic and spectroscopic techniques. For example, SEM has been used along with IR spectroscopy to study the cell wall architecture of Maize coleoptile (Carpita et al., 2001), and with AFM to study different plant tissues like cucumber hypocotyls, *A. thaliana*, and onions (Marga et al., 2005; Xiao and Anderson, 2016; Zhang et al., 2016).

In addition to estimates from real-space images, estimates of microfibril dimensions have been obtained from reciprocal space techniques. These approaches have the advantage of averaging structural features over large areas. Line broadening in X-ray diffraction (XRD, or wide-angle X-ray scattering, WAXS) is directly related to the coherence length *t*, as given by the Scherrer formula:

$$t=\frac{k\lambda }{\beta \,\cos\theta }$$

where λ is the X-ray wavelength, θ is the Bragg angle, *k* is a shape factor that is often 0.89, and β is the angular half width of the line profile. The coherence length is equivalent to the crystal size if fluctuations or defects in the crystal lattice are not cumulative, such that deviations from ideal average lattice positions do not disrupt the long-range order of the lattice. Under this assumption, early applications of this approach measured the cellulose crystallite size for valonia, tunicin, cotton, ramie, *Acetobacter xylinum*, and *Chaetomorpha melagonium* (Nieduszynski and Preston, 1970; Caulfield, 1971). Line broadening of the equatorial reflections (200) and (110/$1\overset{¯}{1}0$ ) give the lateral dimension while the meridional reflection (004) gives the longitudinal dimension. The reported crystal widths from XRD (100–200 Å) significantly exceed values reported for microfibril diameters from electron microscopy (35 Å) and other techniques (see Table 3). One possible explanation is that microfibrils aggregate and strong interactions maintain lattice coherence, thereby leading to apparent larger crystal dimensions from X-ray experiments.

Analyses of XRD data have also attempted to resolve diffraction peaks into Gaussian and Cauchy profiles (Hindeleh and Johnson, 1972, 1974). The obtained crystallite sizes did not support the existence of elementary microfibrils. The results, however, depend a lot on the details of the model adopted for peak fitting, such as the type of fitting function and background subtraction. Other factors like crystal morphology, distortions, and size distribution also affect the results.

In addition to X-ray diffraction, small-angle scattering techniques have also been employed to examine the dimensions of microfibrils. These techniques involve analysis of the intensity of radiation scattered from the sample as a function of the scattering vector *q*. Focusing on small scattering angles can reveal the size and shape of objects, such as the diameter of rod-like microfibrils. Diameters of highly oriented fibrils were obtained from Small Angle X-ray Scattering (SAXS) of ramie, cotton, jute, flax, and cordura using Guinier plots for cylindrical particles (Heyn, 1955). The sizes obtained for jute, ramie, and cotton were in agreement with coherence lengths (crystal sizes) previously obtained from XRD and with diameters obtained from electron microscopy with negative staining (Heyn, 1966). Nevertheless, the weak spatial organization of primary cell walls make interpretation of SAXS profiles challenging; yet, SAXS has successfully been used to examine the size and arrangement of cellulose fibrils in secondary cell walls of spruce wood (*Picea abies*). An almost constant diameter of 2.5 nm with a standard deviation as small as 0.14 nm was found for measurements from 10 different trees (Jakob et al., 1994). This microfibril diameter was in good agreement with that obtained from TEM, which reported the diameter to be 2.4 nm but with a standard deviation as high as 1.3 nm. Other work has demonstrated good agreement between SAXS profiles and Fourier transforms of TEM micrographs (Jakob et al., 1995).

An advantage of SAXS is the ability to perform experiments under moist environments; for example, hydration-dependent structural changes of cellulose microfibrils in spruce wood have been examined (Jakob et al., 1996). The packing density and fibril center-to-center distance was estimated, and it was found that the structure of the cell wall was independent of hydration if the moisture content was above the saturation point of fibrils. Comparable measurements were not possible for moisture content below the saturation point, as the scattering from pores could not be neglected. Similarly, SAXS has been used to study the effect of hydration on cellulose from different sources including *Acetobacter xylinus*, flax, sugi wood, and celery collenchyma (Astley and Donald, 2001; Astley et al., 2001; Suzuki and Kamiyama, 2004; Kennedy et al., 2007b). Such studies are mostly on secondary cell walls as in the case of flax or wood. Celery collenchyma offers a convenient experimental platform for studying hydrated primary cell walls through scattering as they have unusually well oriented microfibrils. It has been reported that hydration increases the mean microfibril spacing from 3.8 nm in dry cell walls to 5.4 nm in hydrated cell walls of celery collenchyma (Kennedy et al., 2007b).

The low scattering contrast between cellulose and other cell wall polymers makes the analysis of X-ray scattering patterns difficult. Small Angle Neutron Scattering (SANS) provides an advantage over SAXS in this context. Because hydrogen scatters much more strongly than deuterium, neutron scattering contrast can be enhanced by replacing H_2_O with D_2_O, or by deuterating components of the cell wall. A SANS study of primary cell walls in celery collenchyma characterized the microfibril diameter and shape (Thomas et al., 2013). The diameter was found to be about 2.9–3.0 nm and this value corresponds to 24 chains in a microfibril with a rectangular cross-section. These results of microfibril diameter and cross-section were similar to the findings of a SANS study of secondary cell wall in spruce wood; nevertheless, the presence of extensive disorder in primary cell walls prevented a conclusive result (Fernandes et al., 2011).

A challenge with scattering approaches is that, in principle, multiple structures can lead to the same scattering profiles. Thus, complementary data is crucial to develop structural models capable of explaining scattering data. This is especially true for primary cell walls, which exhibit poorly ordered packing, and as a consequence, scattering data from these tissues is more challenging to interpret. As such, the application of spectroscopic techniques, such as SS-NMR and IR, to primary cell walls is important to complement scattering and microscopy.

One early report that combined spectroscopy with imaging investigated onion and quince cell walls with fibril diameters established by electron microscopy of 8–10 nm and 2 nm, respectively (Ha et al., 1998). The authors proposed that six microfibrils aggregate in onion, such that each elementary fibril is about 2 nm; a strongly charged hemicellulose coating in quince is proposed to keep these microfibrils isolated. Two independent approaches were adopted for measuring the crystallite diameter, by calculating the proportion of surface to interior chains and through spin-diffusion experiments to measure the distance between surface and interior chains. Altogether, the two methods suggest that fibrils from onion and quince have similar crystallite diameters of approximately 2 nm.

The lateral dimensions of cellulose crystallites from 10 different sources were estimated using ^13^C NMR signal strengths (Newman, 1999). Signals at 89 and 85 ppm were assigned to C4 in the interiors and on the surfaces of crystallites, respectively. Lateral dimensions were estimated from the relative signal areas under an assumption of a square microfibril cross-section. When compared with XRD results of the same samples, lateral dimensions obtained from NMR were found to be 10% higher, and this deviation was attributed to different molecular conformations of surface and interior chains that lead to broadening of XRD peaks. Using the same aforementioned peak assignment of surface and interior chains, NMR was also used to study the microfibril diameter of celery collenchyma and the results compared with that obtained from XRD and SAXS (Kennedy et al., 2007a). Assuming a constant microfibril diameter and circular model for its cross-section, the microfibril radius is calculated as:

$$\frac{A_{1}}{A}=\frac{{\left(R-S\right)}^{2}}{R^{2}}$$

where *A*_I_*/A* is the relative area of signals from interior chains, *R* is the radius, and *S* is the thickness of the surface monolayer of chains calculated from cellulose Iβ lattice parameters as previously reported (Nishiyama et al., 2003). If no structural difference between surface and interior chains is assumed, the size of microfibrils obtained from NMR is in agreement with XRD results. Thus, NMR measurements can reconcile with the entire range of SAXS measurements depending on the different rotational orientation of surface chains that is assumed.

In addition to NMR, IR spectroscopy has been used to extract estimates of the microfibril size in higher plants, algae, and tunicates (Horikawa et al., 2009). This approach is based on an initial deuteration of OH groups in the entire crystalline region followed by re-hydrogenation at 25°C during which deuterated (OD) groups on the surface become re-hydrogenated (OH). Microfibril dimensions were then estimated from the absorbances (*A*) of OD and OH groups. Defining *R* as an empirical parameter that is the ratio of the OD absorbance (*A*_OD_) to the total absorbance by *R* = *A*_OD_/(*A*_OH_ + *A*_OD_) can then enable comparison with other measures of the microfibril diameter. Indeed, *R* was found to be highly correlated to the full width at half maximum of the (200) peak in XRD. Microfibrils were proposed to be flat based on the behavior of the re-hydrogenation process under heat treatment, which was consistent with observations by electron microscopy.

More recently, detailed studies on the cross-sectional shapes of cellulose crystallites and the number of chains in each microfibril have been attempted through spectroscopic techniques. These methods also provide valuable insights into aggregation and twinning of microfibrils, as well as conformational and packing disorder. SS-NMR and IR were used in combination with SANS and XRD to study the microfibril structure of spruce wood (Fernandes et al., 2011). The results of these studies favored a 24-chain model with a rectangular microfibril cross-section and the presence of twisting and disorder that increases toward the surface. Another study on celery collenchyma used NMR and IR of deuterated samples in combination with XRD, SANS, and WANS (neutron diffraction) (Thomas et al., 2013). This study suggests a 24-chain model with eight hydrogen bonded sheets of three chains and also the possibility of an 18-chain model if the presence of a hemicellulose chain is included. It also proposed the presence of high disorder in conformation, packing, and hydrogen bonding. Simulations of XRD profiles were compared with synchrotron XRD data and NMR results to predict the number of chains in microfibrils (Newman et al., 2013). The number of chains in a microfibril was estimated using the crystallinity *x* estimated from NMR spectra (Newman, 1999). The uncertainties involved in the estimation of *k* (shape factor) and *x* made it difficult to make a precise estimate, and a possibility of 17–22 chains was suggested. The study ruled out a 36-chain model on the basis of predicted peaks that did not match with the experimental diffractogram. Good fits were obtained for 24- and 18-chain models, with an even better fit for the 18-chain model with mixed cross-sectional shapes and the presence of occasional twinning.

Furthermore, studies of the cellulose synthase complex suggest a rosette that is a hexamer composed of trimers (Hill et al., 2014; Nixon et al., 2016; Vandavasi et al., 2016), which would be consistent with an 18-chain model. Using this as a starting point, a detailed study that combines X-ray diffraction and NMR data with predictions from computer simulations established a 5-layer cross-section with a 34443 chain arrangement as most probable (Kubicki et al., 2018). The ability to compare predicted and measured ^13^C NMR shifts and diffraction spectra was able to rule out a 6 × 3 arrangement as highly unlikely, although a 6-layer 234432 cross-section is only slightly less likely than the 34443 configuration.

### Cellulose Microfibril Angle

In contrast to the dispersed cellulose orientation of primary cell walls, cellulose microfibrils in woods are wound around the cell in a helical manner whose pitch is defined by the microfibril angle (MFA), which is described as the angle that the microfibrils make with the long axis of the cell (Barnett and Bonham, 2007). Traditionally, the MFA has been used to describe the orientation of cellulose microfibrils in the S2 layer of secondary walls in woods because cellulose makes up the greatest proportion of the wall thickness and most affects the macroscopic physical properties (Senft and Bendetsen, 1985). The S2 MFA has a significant influence on tensile strength, stiffness, and shrinkage in wood (Cave, 1968). Both the longitudinal tensile strength and stiffness of wood have been shown to be markedly affected by MFAs; as the MFA increases, tensile strength and stiffness quickly decrease (Altaner and Jarvis, 2008). The MFA is also an important determinant of quality of wood products. It has a major effect on the stability of wood on drying and subsequent manufacturing processes (Zobel, 1961).

The techniques for measuring MFAs can be grouped into four categories: (1) Polarized light microscopy, (2) direct visualization through microscopy after physical or chemical treatment such as iodine staining, (3) XRD and SAXS, and (4) Near IR (NIR) spectroscopy. A detailed review of these techniques and their comparison is available (Donaldson, 2008), and a brief summary of results from various techniques is shown in Table 4.

**Table 4:** Microfibril angle from different sources of cellulose obtained through the use of different characterization techniques.

Source of cellulose	Microfibril angle (^o^)	Techniques^∗^
*Picea abies*	≤5 (earlywood), 20 (latewood)	SAXS (Jakob et al., 1994)
	8 (earlywood), 9 (latewood)	XRD (Sahlberg et al., 1997)
*Picea excelsa*	32—35 (normal wood)	XRD (Kantola and Seitsonen, 1961)
	39—43 (compression wood)	
	18 (normal wood)	SAXS (Kantola and Kähkönen, 1963)
	25—45 (compression wood)	
Cedar (branch)	39—57	PLM (Preston, 1934)
Japanese larch	37—79	PLM (Preston, 1934)
Abies nobilis	23—69	PLM (Preston, 1934)
Virginia pine	20	PLM (Mark, 1967)
Loblolly pine	4—25 (latewood)	SM (Hiller, 1964)
	19.22—34.06	NIR (Jones et al., 2005)
Slash pine	10—40 (latewood)	SM (Hiller, 1964)
Douglas fir	20 (early &and normal wood)	XRD (El-osta et al., 1973)
	7—30	PLM (Erickson and Arima, 1974)
Pinus radiata	10.7—41.6	NIR (Schimleck et al., 2002)
	12—27	PLM (Boyd and Foster, 1974)


**^∗^PLM:, polarized light microscopy; SM:, staining methods; NIR:, near IR spectroscopy; XRD:, X-ray diffraction; SAXS:, small angle X-ray scattering.**

Extracting MFAs from polarized light microscopy involves rotating cellulose fibers relative to the fiber long axis until the maximum extinction position (MEP) is reached, which occurs when the bright cell wall becomes dark (Preston, 1934; Page, 1969). The difference between the fiber axis and MEP gives an estimate of an average MFA. A disadvantage of this technique is that it requires samples consisting of a single cell wall, otherwise the orientation of microfibrils in opposing cell walls in front and back walls will inhibit accurate determination of the MEP (El-Hosseiny and Page, 1973).

Brightfield microscopy and confocal microscopy have been used to measure MFAs in iodine stained samples (Bailey and Vestal, 1937; Senft and Bendetsen, 1985; Donaldson and Frankland, 2004). This method involves precipitation of iodine crystals within the cell wall and hence, it is limited by the fact that not all wood samples react well with iodine; thus, iodine does not always uniformly disperse in all the cells. Because iodine sublimes fast, the measurements have to be taken rapidly. Higher accuracy measurements of MFAs were facilitated through high contrast images taken with confocal reflectance microscopy (Donaldson and Frankland, 2004) or electron microscopy (Wardrop and Preston, 1947; Frei et al., 1957; Dunning, 1968).

X-ray diffraction is perhaps the most commonly used method for determination of MFAs. Typically, MFA is obtained from XRD through the azimuthal distribution of the cellulose (200) equatorial reflection (Cave, 1968; Nelmes and Preston, 1968; Yamamoto et al., 1993). This method assumes that the cellulose crystals do not have a preferred orientation around the microfibril axis. SAXS can also provide MFA in a similar manner as XRD without this assumption (Jakob et al., 1994; Reiterer et al., 1998). SAXS has been used to estimate MFA in primary cell walls of single celled alga *Chara corallina* and multicellular hypocotyl of *A. thaliana* (Saxe et al., 2014). The work shows a bimodal MFA distribution such that the bulk of the microfibrils are oriented either transversely or longitudinally with broad scattering. The highly oriented microfibrils in secondary walls give an anisotropic SAXS pattern and the azimuthal intensity distribution of the resulting streaks is used to extract information on the distribution of MFA. This method has been adopted for wood cells in *Picea abies* (Jakob et al., 1994; Reiterer et al., 1998). These studies found that stiffer parts of trees have lower MFA when compared to the more flexible parts that have higher MFA, thereby supporting the correlation between cellulose MFA and mechanical properties of the cell wall.

Near IR spectroscopy has also been used to predict MFA by examining wood surfaces on the radial-longitudinal face (Jones et al., 2005; Schimleck et al., 2005). The method uses XRD data for calibration, and thus becomes inaccurate for higher angles because XRD data are less precise at high angles due to a reduced signal-to-noise ratio for the (200) reflection of the diffraction pattern (Schimleck et al., 2005).

### Spatial Organization of Cellulose Microfibrils

Because cellulose microfibrils are the structural units of primary cell walls, the spatial arrangement of these microfibrils, including their bundling and packing, strongly impacts cell wall mechanics and growth. Traditionally, the mesoscale arrangement of microfibrils was studied largely by electron microscopy. The technique provided many valuable insights about the microstructure in cell walls, such as the development of network-like morphologies in growing cells of maize and oats coleoptiles (Mühlethaler, 1950). Microfibrils form a loosely reticulated network in a newly deposited cell wall, and gradually stiffen the wall with the addition of new microfibrils. Electron microscopy has also been used to study the cell wall architecture of near native onion primary cell walls at high resolution through shadowed replicas of rapidly frozen, deep-etched specimens (McCann et al., 1990). This study suggests hemicelluloses form the cross-links between cellulose microfibrils, and indicated a lamellate model for cellulose organization; microfibrils are co-aligned within each “lamellae,” multiple lamellae (ca. 100) are stacked on top of each other, but the net orientation of each lamellae is not necessarily correlated to other lamellae. Various aspects of this model were challenged by further work on native tissues, as described below.

**FIGURE 7:**
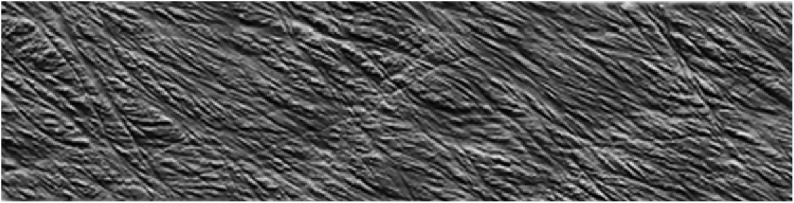
Atomic Force Microscopy micrograph of cellulose microfibrils merging in and out of microfibril bundles. Reprinted with permission from Zhang et al. (2016). Spatial organization of cellulose microfibrils and matrix polysaccharides in primary plant cell walls as imaged by multichannel atomic force microscopy. The Plant Journal 85, 179–192. Copyright © 2016, John Wiley and Sons.

Although limited to the structure near the surface, SEM and AFM provide an opportunity to image the spatial arrangement of microfibrils in primary cell walls. SEM has been demonstrated as a powerful tool to examine microfibril organization and will be discussed in more detail in the next section in the context of examining the interaction between cell wall components; AFM provides a relatively unique capability of imaging cell walls in their native state. For example, detailed observations of the primary cell walls of onion and *Arabidopsis* have elucidated multiple aspects of the cellulose network structure. Contrary to reports based on electron microscopy (McCann et al., 1990), high resolution images of microfibrils in their native state for onion did not support the hypothesis of microfibrils cross-linked by hemicellulose. Instead, AFM images show microfibril bundles with single microfibrils emerging in and out to form a reticulated network (Zhang et al., 2014, 2016). Figure 7 shows a montage of high resolution AFM images of onion where the alignment of microfibrils and extensive microfibril bundling is visible. Often, multiple layers are visible, such that the relative orientation of the layers can be examined. The studies suggest a crossed polylamellate wall structure instead of a helicoidal arrangement.

**FIGURE 8:**
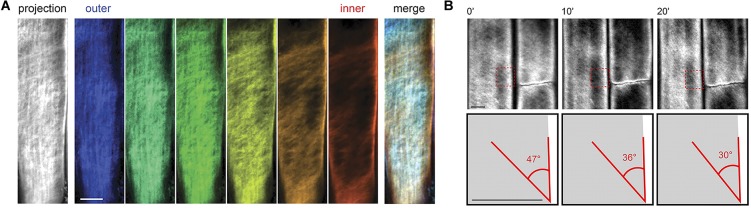
Confocal microscopy images showing **(A)** cellulose orientation in different cell wall layers using S4B staining, and **(B)** rotation of stained microfibrils over time in *Arabidopsis*. Reprinted with permission from Anderson et al. (2010). Real-Time Imaging of Cellulose Reorientation during Cell Wall Expansion in *Arabidopsis* Roots. Plant Physiology 152 (2), 787–796. www.plantphysiol.org. Copyright © 2010 American Society of Plant Biologists.

As a complementary technique to AFM, fluorescence microscopy can characterize cellulose microfibrils with high sensitivity and selectivity to chosen markers despite low spatial resolution (∼200 nm). Xyloglucan binding proteins, galactan-binding proteins, or antibodies have been used with fluorescent labels for visualizing the distribution of hemicellulosic components in cell walls (Hayashi and Maclachlan, 1984; Brunecky et al., 2008; Sandquist et al., 2010). Nevertheless, the large size of these proteins restricts penetration into interstitial spaces and nano-sized pores within the cell wall structure. The search for smaller probes led to the discovery of Carbohydrate Binding Modules (CBM) as suitable molecular probes for high-resolution fluorescence microscopy because of their compact size and specificity toward targeted substrates. According to their substrate specificity, CBMs are classified as Types A, B, and C, where Type A binds to the surface of crystalline polysaccharides, B binds internally to glycan chains, and C binds to termini of glycan chains (Gilbert et al., 2013). Fluorescence microscopy with CBMs as molecular probes has been used to investigate the structure of cellulosic material both in native and treated samples (Porter Stephanie et al., 2007; Kawakubo et al., 2009; Široký et al., 2016). In addition, confocal microscopy with the fluorescent dye Pontamine Fast Scarlet 4B (S4B), a stain that shows higher specificity for cellulose than for other cell wall components, has been used to study the cell wall architecture and dynamics of cellulose microfibrils in growing cell walls of *A. thaliana* root cells (Anderson et al., 2010). Confocal fluorescence microscopy images from this study supported the passive reorientation theory of cellulose microfibrils, which states that newly deposited cellulose microfibrils are transversely oriented to the longitudinal axis and the microfibrils reorient during expansion. Figure 8 shows confocal images of cellulose orientation in different cell wall layers using the S4B stain. As a function of time, the cellulose microfibrils reorient from approximately 47–30° with respect to the long axis of the epidermal cells.

Scattering methods again provide a complementary approach to microscopy. Ultra-small angle (USAXS) and very small-angle X-ray scattering (VSAXS) are being used with SAXS to study the hierarchical structure of cellulose. USAXS can probe length scales from 1 to 10 μm, thus enabling the study of microfibril bundles or aggregates, while VSAXS can probe length scales intermediate between that of SAXS and USAXS. The scattering patterns of untreated and pre-treated maize using these techniques reveal the presence of structures with sizes in between microfibrils of 30 nm diameter (likely microfibril aggregates) and 140 nm bundles (Inouye et al., 2014; Zhang et al., 2015). Yet, details regarding the origin of these scattering features remain elusive.

Another approach to examine the spatial arrangement of cell walls is based on SFG. The non-centrosymmetry and phase matching requirements and the coherence length on the order of hundreds of nanometers lead to signatures of the spatial organization of crystalline cellulose dispersed in amorphous matrices. In particular, the overall SFG intensity, the alkyl peak shape, and the alkyl/hydroxyl intensity ratio have been shown to depend on the mesoscale assembly of cellulose, such as the lateral packing and net directionality of microfibrils (Lee et al., 2014). Recent work shows that SFG can detect the difference in arrangement of cellulose microfibrils between primary and secondary cell walls (Lee et al., 2014, 2015a). On the basis of the CH/OH relative intensity in SFG, it was suggested that over the SFG coherence length, primary cell walls have a lower degree of antiparallel orientation of cellulose microfibrils (Lee et al., 2014). Furthermore, control samples with uniaxially aligned cellulose crystals in amorphous matrices were examined to identify spectral signatures corresponding to the distance between microfibrils, and these signatures are supported with predictions of the spectra. The work on these model systems suggests that the CH/OH intensity ratio in SFG spectra decreases non-linearly as the intercrystallite distance increases (Makarem et al., 2017). In addition, because SFG can be performed on hydrated samples, the effect of drying has been examined. Reversible changes in the SFG spectra with dehydration and rehydration were attributed to the presence of local strains due to drying (Huang et al., 2018). The consequence of such strains could be to perturb the packing of cellulose, thereby affecting the width and position of diffraction peaks. Further work is needed to determine the consequences of drying, and to ensure that X-ray and electron beam techniques that rely on dry samples yield reliable and biologically relevant structural information.

The aforementioned techniques provide valuable insights into the arrangement of cellulose microfibrils in cell walls. Nevertheless, relating cell wall structure with cell growth and mechanics requires an understanding of the interaction of cellulose with other matrix polysaccharides. The different approaches and techniques focused in this area are discussed in the following section.

## Interaction of Cellulose Microfibrils With Other Matrix Polysaccharides

Cell wall properties are dependent upon the combined structure, chemistry, and mechanical properties of the constituents (Chebli and Geitmann, 2017). Cellulose–cellulose and cellulose-matrix interactions influence the strength and extensibility of cell walls, thus contributing to the regulation of cell growth. The major non-cellulosic polymers in primary walls are different from those in secondary walls (Cosgrove and Jarvis, 2012). Xyloglucans and pectin are dominant in primary walls, and the current structural model of the primary wall depicts a cellulose-hemicellulose network embedded in a pectin matrix. These constituents form the crucial load bearing components. In secondary walls of coniferous wood, cellulose microfibrils form aggregates with adjacent microfibrils directly attached to each other over part of their length, and most of the hemicellulose and lignin lie out of these aggregates, with glucomannans more closely associated with the microfibrils (Fernandes et al., 2011). These structural models were derived from chemical analysis, biochemical studies, and electron and optical microscopies (Carpita and Gibeaut, 1993; Sarkar et al., 2009). New approaches to examine the interaction of cellulose and matrix polysaccharides involve scattering, spectroscopy, and microscopic techniques, such as AFM and FESEM. The following section discusses the application of these techniques to investigate interactions between cellulose and matrix polysaccharides.

The heterogeneity of the cell wall composition complicates the application of characterization techniques to the whole cell wall. Methods to isolate interactions of specific wall components can be roughly classified in one of two ways: (i) top-down approaches and (ii) bottom-up approaches (Martínez-Sanz et al., 2015a). The top-down approach involves investigating the effects of removal of non-cellulosic components on the structure of the cell wall, while the bottom-up approach involves the incorporation of additives into the culture media of cellulose-producing bacteria to mimic the assembly process taking place during plant cell wall biosynthesis. Bottom-up approaches are limited in relevance to primary cell walls given that a detailed description of cell wall assembly is currently not available; nevertheless, such studies are potentially informative as we learn more about cell wall structure and assembly and we thus briefly discuss them here.

**FIGURE 9:**
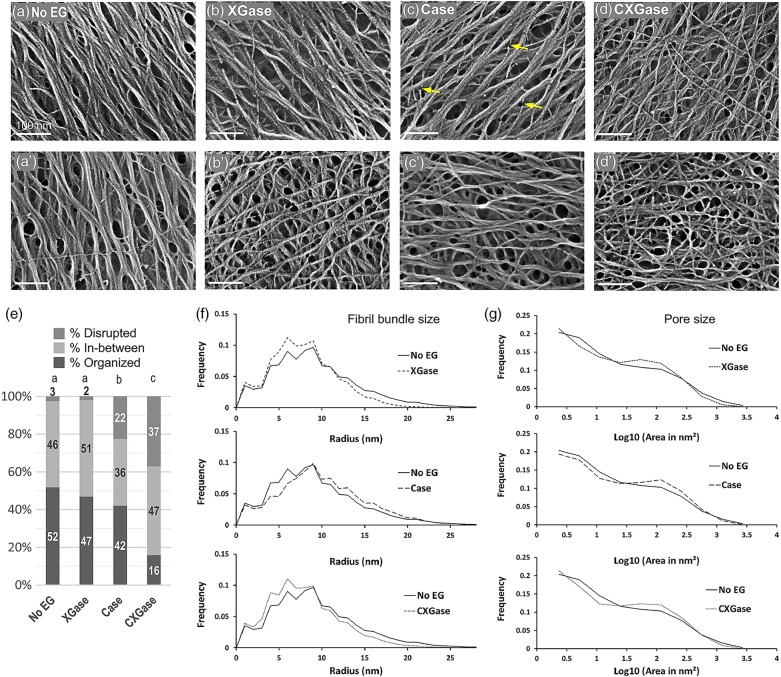
Field emission scanning electron microscopy (FESEM) images of onion cell wall without enzyme treatment **(a,a’)** and after different enzyme treatments **(b–d,b’–d’)**. **(e–g)** Effect of treatments on wall properties. XGase is a family-12 glycosyl hydrolase (GH) that hydrolyzes xyloglucan but not unbranched glucan, Case is a family-12 GH that hydrolyzes non-crystalline cellulose only, and CXGase is a family-5 GH that hydrolyzes both non-crystalline cellulose and xyloglucan. No EG denotes no endoglucanase treatment. Reprinted with permission from Zheng et al. (2018). Xyloglucan in the primary cell wall: assessment by FESEM, selective enzyme digestions and nanogold affinity tags. The Plant Journal 93, 211–226. Copyright 2018, John Wiley and Sons.

In the top-down approach, non-cellulosic components of the cell wall can be removed by techniques including enzymatic hydrolysis and acid hydrolysis (Pingali et al., 2010), and by treatment with base (Jungnikl et al., 2007), steam (Pingali et al., 2014), or ionic liquids (Cheng et al., 2011). The effects of enzymatic hydrolysis on the structure of the cellulose network have been widely studied by SAXS and SANS (Kent et al., 2010; Penttilä et al., 2010, 2013). These studies suggest that hydrolytic digestion proceeds from the outer surface and very often cannot penetrate into the substrate interior without agitation of the sample. In addition, SEM and TEM have been extensively used to follow structural changes in the cell wall after biomass pre-treatment (Sant’Anna and de Souza, 2012). SEM is the method of choice to describe anatomical features and degradation at cellular- and nano-resolution of biomass surfaces, while TEM is combined with techniques including ultra-thin sectioning, rapid-freezing followed by deep etching, ultrastructural cytochemistry, immunogold, and electron tomography to investigate ultrastructural changes in the cell wall. In a recent study, FESEM was used to investigate fiber bundling, organization, and the spatial location and conformation of xyloglucans in onion cell walls (Zheng et al., 2018). FESEM imaging was combined with digestions by substrate-specific endoglucanases and labeling with nanogold affinity tags for cellulose and xyloglucan (Figure 9). The study provided evidence of coverage of cellulose surfaces by xyloglucan to some extent, but distinct xyloglucan structures could not be imaged. In particular, a lack of evidence for xyloglucan tethered to multiple microfibrils suggests xyloglucan does not serve as load-bearing links between microfibrils.

Atomic force microscopy has also been applied to study the effect of chemical extraction procedures on the structure of cellulose microfibrils (Davies and Harris, 2003; Kirby et al., 2006). In a study of the effect of thermochemical treatment on maize cell wall (Chundawat et al., 2011), the ability of the AFM tip to differentiate between hydrophobic and hydrophilic regions was used to reveal that the native cell wall is mostly hydrophobic. Nevertheless, after thermochemical treatment, hydrophilic regions were found. An increased surface roughness could also be measured by AFM.

Alternatively to extraction, mutants have been used to examine the effects of modifying cell wall compositions and reveal interactions between cellulose and matrix components. Xyloglucan deficient mutants of *A. thaliana* (*xxt1 xxt2*) show highly aligned cellulose microfibrils in AFM images of the cell wall (Xiao et al., 2016). This increase in local order suggests that xyloglucan mediates interactions between cellulose microfibrils, as a spacer molecule that promotes microfibril dispersion within the cell wall. Pectin mutants of *A. thaliana* (*PGX1^AT^*) lead to shorter homogalacturonan, and ^13^C solid-state NMR reveals perturbations to the pectin-rich matrix and pectin-cellulose interactions in the cell walls of these plants (Phyo et al., 2017). The overall larger growth of pectin mutants and ^13^C NMR characterization suggests that the pectin matrix influences wall dynamics during cell growth. The on-going studies of mutants will continue to reveal fundamental interactions between cell wall components.

Another approach relies on labeling of components to provide sensitivity to specific interactions. Multidimensional solid-state NMR (MAS SS-NMR) spectroscopy, coupled with ^13^C labeling of whole plants, enabled study of the spatial arrangement of cell wall polysaccharides in near-native cell walls. The analyses of cross-peaks in two- and three-dimensional MAS SS-NMR of ^13^C labeled *A. thaliana* suggests that cellulose forms a single network with pectin and xyloglucans (Wang and Hong, 2016). The technique also revealed the existence of pectin-cellulose close contacts in primary cell walls (Wang et al., 2012). ^13^C SS-NMR of mung bean cell walls detected xyloglucans of different mobilities including rigid and partly rigid (Bootten et al., 2004); the study suggests that the partly rigid xyloglucans are predominant in the cell wall. In addition, polarization transfer in SS-NMR has been used to study water-polysaccharide interactions in primary cell walls of *Arabidopsis.* Results on water-pectin and water-cellulose spin diffusion support the single network model of the primary cell wall (White et al., 2014). Furthermore, MAS NMR of *Arabidopsis* stems revealed that xylans are found in both two and threefold screw conformations (Simmons et al., 2016). The twofold conformation is required for xylans to bind onto cellulose microfibrils.

Bottom-up approaches use a cellulose-producing bacteria such as *Gluconacetobacter xylinus* as a model system for the study of cellulose-matrix polysaccharide interactions. Cell wall polysaccharides like hemicellulose and pectin are incorporated into the culture media of the bacteria and composite pellicles are produced. The bacterial cellulose composites can then be used to examine how matrix polymers affect cellulose crystallization and how cellulose interacts with matrix polysaccharides. For example, XRD, SAXS, and SANS have been used widely to study composite pellicles with cell wall polysaccharides including xylan, xyloglucan, arabinoxylan, mannan, and pectin (Astley et al., 2001; Gu and Catchmark, 2012; Martínez-Sanz et al., 2015b). SAXS and XRD studies revealed that addition of xyloglucan affects the cellulose microfibril packing and crystalline structure; in contrast, addition of arabinoxylan does not impact these features of the cellulose network (Martínez-Sanz et al., 2015b). Spectroscopy and microscopy can also be used to examine the cellulose network within composite pellicles; nevertheless, the pellicles are highly hydrated and have strong aggregation tendency, so structural artifacts may be introduced during the drying process that is required for analysis. Recent SANS studies demonstrated that controlled incorporation of deuterium into bacterial cellulose does not introduce any structural changes in bacterial cellulose (Bali et al., 2013; He et al., 2014). The deuterated bacterial cellulose will have applications in elastic and inelastic neutron scattering experiments for studying cellulose structure and dynamics and interactions with wall polysaccharides.

Similar to scattering techniques, spectroscopic techniques including IR, Raman, SFG, and NMR have also been used to investigate interaction among cell wall components through both top-down and bottom-up approaches. IR spectroscopy has been used to study the changes in cellulose polymorphism on addition of xyloglucan, xylan, arabinogalactan, and pectin to bacterial cellulose (Tokoh et al., 1998, 2002; Gu and Catchmark, 2012). Addition of xylan and xyloglucan results in an increase in the levels of cellulose Iβ and a decrease in crystallinity. Xyloglucan has a larger impact on cellulose assembly than pectin as addition of xyloglucan decreases crystallinity and increases disorder in the cellulose structure, but addition of pectin has no effect (Gu and Catchmark, 2012).

## Opportunities in Structural Characterization of Plant Cell Walls

The on-going development of instrumentation and techniques for the study of soft matter structure leads to new opportunities in the structural characterization of cell walls. In this section, we highlight some emerging techniques based on diffraction/scattering, imaging, and spectroscopy that may facilitate the creation of new knowledge on cell wall structure and assembly.

### Diffraction and Scattering

The high brilliance of synchrotron radiation sources has enabled the application of X-ray microbeam diffraction and scattering techniques to weakly scattering samples like polymers and biopolymers. X-ray diffraction and scattering techniques can provide average structural parameters, but not information on local structures. Beam sizes of about 1 μm and sub-μm sizes can provide abundant local information, such as the spatial heterogeneity of materials and the structural change at a local position. An advantage of scanning X-ray diffractometry when compared to transmission electron scattering experiments is the ability to examine single fibers without the necessity for sectioning (Riekel, 2000).

The application of position-resolved synchrotron X-ray microdiffraction with beam size less than the thickness of a single cell wall enabled the imaging of the helical arrangement of cellulose microfibrils in cell walls of Norwegian spruce (Lichtenegger et al., 1999; Peura et al., 2005). X-ray microbeam diffraction has also been used to study the orientation, crystallite size, and crystallinity of cellulose microfibrils from various sources, including viscose rayon fibers (Müller et al., 2000), Japanese Cedar (Müller et al., 2002), and Norway spruce (Peura et al., 2007). For techniques aiming at analyzing small sample volumes, X-ray microdiffraction has a clear advantage over transmission electron microscopy/diffraction in terms of sample preparation and acquisition time. The application of SAXS with a beam size of a few micrometers (μSAXS) revealed the strong alignment of cellulose microfibrils within single native flax fibers (Müller et al., 1998). Such position-resolved studies could potentially resolve the super-molecular structure of cellulose microfibrils.

Grazing Incidence Wide Angle X-ray Scattering (GIWAXS) is another synchrotron based technique (although it is becoming available in lab-scale instruments) that may be useful for primary plant cell walls. GIWAXS probes not only the surface but also beneath it. Because of its grazing incidence geometry, GIWAXS is a promising scattering technique for weakly scattering and fragile cell wall samples. The large beam footprint produces a better signal-to-noise ratio and also causes less radiation damage. GIWAXS with a 2D detector can reveal net orientation of crystals, ca lled texturing (Baker et al., 2010 #690; Gomez et al., 2011 #1026; Rivnay et al., 2012 #3971). GIWAXS data from a cell wall sample can be used to estimate the degree of preferred orientation and crystallinity of cellulose crystals, which has not been previously demonstrated.

Resonant soft X-ray scattering (RSoXS) is a combination of conventional SAXS with soft X-ray spectroscopy that offers enhanced and tunable scattering contrast as well as elemental and chemical environment sensitivity (Virgili et al., 2007; Guo et al., 2013; Liu et al., 2016). Its large length scale accessibility, chemical sensitivity, and molecular bond orientation sensitivity makes RSoXS an attractive tool for studying different materials including biological assemblies. The different cell wall polysaccharides have similar electron density, so RSoXS could be useful in differentiating between them based on their chemical differences. Recent work has shown that RSoXS can reveal the structure of casein micelles and proteins by tuning to specific X-ray energies and thereby producing contrast between components (Ingham et al., 2015, 2016; Ye et al., 2018b). Furthermore, work on onion scales has demonstrated that tuning the X-ray energy to the Ca edge generates contrast between pectin and cellulose microfibrils, such that the spacing between microfibrils or microfibril bundles is revealed (Ye et al., 2018a). Thus, an opportunity exists to adopt a new chemically sensitive scattering technique for the study of plant cell walls.

In addition to X-ray scattering, there are opportunities for novel characterization approaches based on neutron scattering. Quasi-elastic neutron scattering (QENS) is sensitive to reorganization of atoms and molecules on a pico-second to nano-second time scale over length scales of 1–500 Å. This broad spatial and temporal scale is ideal for studying complex biological systems as the scale is matched to atomic and molecular vibrational displacements, jump distances, and correlation lengths (Magazù and Migliardo, 2011). Because of the dependence of the relaxation times on the wave-vector, QENS can resolve spatial differences in the dynamics of water and biological macromolecules like proteins. The technique has also been applied to study water-cellulose dynamics in bacterial cellulose, which revealed the existence of two distinct populations of water in the bacterial cellulose system: surface water and water confined in the spaces between the microfibrils(O’Neill et al., 2017). Even though the nanoscale structure and composition of bacterial cellulose is markedly different from plant cell walls, the feasibility of the study presents the technique as a promising tool for the study of native plant cell walls as well.

### Microscopy

Recent advances in optical, X-ray, and electron imaging tools provide new opportunities for the study of cell walls. Optical microscopes cannot distinguish between two objects separated by a lateral distance less than approximately half the wavelength of light used to image the specimen. This resolution limitation is referred to as the diffraction limit. The diffraction limit for optical microscopy is about 200–300 nm in the lateral direction and 500–700 nm in the axial (vertical) direction for confocal microscopy, which makes subcellular structures too small to be resolved in detail. This presents a problem when optical microscopy is used to investigate plant cell wall features of about a few nanometers in size. In such cases, the signal collected by optical microscopy represents an ensemble average of signals from different wall constituents. Super Resolution Fluorescence Microscopy (SRFM) refers to a host of techniques that overcome the resolution limitation caused by the diffraction limit in conventional fluorescence microscopy (Huang et al., 2009). With SRFM, three-dimensional imaging with an optical resolution of about 20 nm in the lateral direction and 40–50 nm in the axial dimension has been achieved. These techniques can employ non-linear optical effects to reduce the size of the excitation point spread function through Stimulated Emission-Depletion (STED) or Saturated Structured Illumination microscopy (SSIM). Furthermore, some techniques are also based on the localization of individual fluorescent molecules, such as stochastic optical reconstruction microscopy (STORM), photoactivated localization microscopy (PALM), and fluorescence photoactivation localization microscopy (FPALM). Recent advances have enabled 3D imaging (Huang et al., 2008), multicolor imaging (Bossi et al., 2008), and live cell imaging (Westphal et al., 2007) with SRFM.

Another approach to increase the spatial resolution beyond the diffraction barrier relies on combining near-field optical techniques with scanning probe microscopy. Near-field scanning optical microscopy (NSOM) obtains high optical and spatial resolution through the use of a tapered optical fiber with a sub-wavelength aperture of about 100 nm in diameter. Because these tips are made from optical fibers, they are fragile and easily damaged, which can lead to artifacts. Due to these issues, NSOM with aperture-less probes with tip enhancement are being used to compartmentalize signals collected from the near field and the far field (Fragola et al., 2004). Such an ability holds promise for characterization of plant cell walls as signals from different cell wall components could be differentiated. Other apertureless tip-enhanced imaging techniques that may be able to chemically characterize plant cell walls on the cellular scale are Tip-enhanced Raman imaging, Near-field coherent anti-stokes Raman Scattering microscopy and two-photon excitation fluorescence (TPEF) spectroscopy. The capabilities of these techniques have been discussed in detail in a review (Yarbrough et al., 2009).

Total Internal Reflection Fluorescence Microscopy (TIRFM) is well suited for optical sectioning at cell-substrate regions with a thin region of fluorescence excitation (Axelrod, 2001; Mattheyses et al., 2010). The laser beam is incident on the glass-substrate interface at an angle beyond the critical angle. Due to the nature of the evanescent field, the excitation volume is large in the transverse dimension but highly confined in the axial dimension. This greatly reduces background fluorescence from out-of-focus planes and results in images with a very high signal-to-noise ratio. TIRFM has proven to be a powerful approach for examination of animal cells and for single-molecule experiments. It is particularly useful for analysis of dynamics of molecules and processes near the plasma membrane as it obscures the fluorescence from the bulk of the cell. Indeed, a recent study applied TIRFM to examine protein endocytosis in the plant plasma membrane (Johnson and Vert, 2017); however, application of TIRFM to the cell wall which lies adjacent to the plasma membrane has not yet been demonstrated. Furthermore, the use of multi-angle TIRFM opens the possibility of examining the distribution of proteins within plant samples in the axial direction (Fu et al., 2016).

High resolution can also be achieved using short-wavelength radiation. Scanning Transmission X-ray Microscopy (STXM) can generate microscopic images of a thin section of a specimen by raster-scanning a focused X-ray beam while the transmitted X-ray intensity is recorded as a function of the sample position. This technique falls under the category of ‘spectro-microscopy’ as X-ray absorption spectra can be obtained from microscopic features of the sectioned sample. The technique is based on synchrotron radiation and leverages X-ray absorption spectra that are characteristic of chemical states of atomic species or crystalline structures of materials (Warwick et al., 1998). Thus, STXM is useful for elemental identification and spatial mapping of heterogeneous materials (Ade and Hitchcock, 2008). The main advantages of STXM are minimal radiation damage (when compared to electron microscopy), ability to analyze hydrated samples, and ability to probe alignment of molecular orbitals due to polarization dependence. Various STXM based techniques like C-XANES and C-NEXAFS have been used to carry out chemical analysis of plant biomass (Cody, 2000; Mancosky et al., 2005; Cody et al., 2009). STXM based spectrotomography is also able to do morphological 3D visualization and quantitative chemical mapping in bacteria (Wang et al., 2011).

STXM based on soft X-ray spectromicroscopy is a powerful technique that holds promise for characterization of plant samples with advantages of high spatial resolution and chemical sensitivity similar to mid infrared spectromicroscopy. The major problem in characterizing cell wall samples is the heterogeneous matrix and the spectra obtained are often dominated by the component in highest concentration. The use of X-ray fluorescent probes can be used with high resolution STXM to overcome the limitations of molecular sensitivity. Using the combination of confocal laser microscopy with fluorescent probes and STXM could be a valuable approach for studying plant cell wall samples. The approach has been demonstrated successfully in microbial biofilms (Lawrence et al., 2003).

Advances in TEM, and in particular in cryogenic transmission electron microscopy (cryo-TEM), provides new opportunities that are also based on short-wavelength radiation. Recent advances in direct electron detectors and automatic image acquisition have significantly advanced structural biology, as these instrumental developments allow for better signal to noise and acquisition of large data sets that can be averaged to improve resolution. The process begins with vitrification, in which the sample solution is rapidly cooled and water molecules form an amorphous solid instead of crystallizing. Resolutions of approximately 3 Å or lower have been achieved by cryo-TEM (Bartesaghi et al., 2015; Dellisanti, 2015). The technique can analyze large and complex biological assemblies that are often difficult to crystallize for X-ray crystallography or are too large and complex for NMR. 3D images of samples can be reconstructed from tilted 2D images through cryogenic electron tomography (cryo-ET). Both cryo-TEM and cryo-ET hold promise for characterization of plant cell walls as they allow analysis of the preserved hydrated state. The use of cryo-TEM to study the cell wall organization of *Staphylococcus aureus* has been demonstrated (Matias and Beveridge, 2006). Cryo-ET has also been used for 3D visualization of cell wall ultrastructure at a resolution of about 2 nm without isolation of cell walls (Sarkar et al., 2014). The microfibril diameter within *Arabidopsis* cell walls found from the study was comparable to diameters measured from AFM. Nevertheless, the sample preparation required for this approach is lengthy and arduous when compared to the more commonly used imaging techniques like AFM and FESEM. Application of faster sample preparation protocols might contribute to more routine use of the technique.

The use of ionizing radiation, as in X-ray or electron microscopy, is limited by the damage caused from the beam. An alternative approach is Scanning Acoustic Microscopy (SAM), which makes use of acoustic waves to create images of microscopic objects. Unlike optical microscopy, SAM does not require any staining or fixation, so it can be used for imaging live cells. Also, it can non-invasively observe not only the surface but also the internal structure of the specimen with sub-micron resolution. In addition, SAM is capable of measuring mechanical properties like the loss factor and modulus of tissues (Maeva et al., 2009). The interactions between ultrasonic waves and matter determine the size of the receiving signal and thus create contrast; contrast is generated on the basis of different acoustic impedances of different materials and is also due to absorption of acoustic waves in the material. Conventional SAM operates in the range of 20–200 MHz while High Frequency SAM (HF-SAM) operates in the 0.4–2 GHz range. HF-SAM has been used to study the hydrated primary cell wall of onion epidermis (Tittmann and Xi, 2014). In this study, SAM was able to detect that enzymatic removal of pectin influences the mechanical properties of primary cell wall. Thus, SAM presents potential as a powerful tool to study not only the structure and mechanics of the cell wall in its natural state but also the interactions between the different wall components through the top-down approach of enzymatic treatments.

As discussed earlier, microscopic techniques are widely used for direct visualization of plant cell walls. Nevertheless, only a few examples of quantitative image analysis have been reported. Typical image analysis includes determining particle sizes, area, length, porosity, and other useful measurements. The availability of open source and open architecture image processing software like ImageJ (Schneider et al., 2012) has contributed to the ability to readily quantify various parameters from microscopic images. For example, ImageJ has been used to process and analyze AFM images to quantify different cellulose microfibril parameters like width and orientation (Boudaoud et al., 2014; Kafle et al., 2014; Zhang et al., 2016, 2017). Several open source image analysis software packages including SOAX (Xu et al., 2015; Zhang et al., 2017) and FibreApp (Usov and Mezzenga, 2015) in addition to ImageJ offer immense opportunities to be used for quantitative analyses of microscopic images of cell walls.

Yet, quantitative analysis of microscopy images of the cell wall is still challenging as the structure is highly heterogeneous. It is even more difficult in primary cell walls due to the higher degree of disorder. A number of times, the arrangement of microfibrils have been reported to mimic a ‘liquid crystal’ like structure (Reis et al., 1991; Himmel et al., 2007). The molecules in such structures seem to have a certain degree of preferred orientation. The amount of ‘order’ in such states can be defined by an order parameter that describes ordering in liquid crystals (Nishiguchi et al., 2017). Currently available image analysis tools have capabilities that enable estimation of such order from microscopy images of cell walls.

### Spectroscopy

The region of the electromagnetic spectrum from 0.1 to 10 THz (3.3–333.6 cm^-1^) is described as the terahertz (THz) region. THz spectroscopy has the ability to distinguish between samples with good and poor long-range order and thus can probe the crystallinity of materials (McIntosh et al., 2012). For THz radiation, crystalline materials present well-defined absorption peaks while amorphous phases present featureless spectra. It can differentiate between different crystalline phases as well (Strachan et al., 2005). THz-time domain spectroscopy (TDS) has been applied to determine the degree of crystallinity of microcrystalline cellulose samples (Vieira and Pasquini, 2014). Because THz radiation is responsible for long-range periodic vibrations in crystals, the absorption bands can be directly related to the degree of crystallinity of the sample. As such, the technique can selectively detect crystalline cellulose and holds promise for characterization of cellulose in native cell wall samples.

Atomic force microscopy-based infrared (AFM-IR) spectroscopy combines the spatial resolution of AFM with the chemical analysis capability of IR spectroscopy. It was developed to overcome the diffraction barrier limitation of IR spectroscopy and the inability of AFM to discriminate materials on the basis of chemical composition. The nanometer scale spatial resolution of AFM-IR allows IR microspectroscopy to investigate many life science problems like subcellular imaging and spectroscopy of bacterial and mammalian cells. Extension of this technique to plant cell wall studies may reveal important information about the spatial distribution of various cell wall components.

## Conclusion and Outlook

Describing the structure of cellulose has direct implications on understanding the anisotropic growth and mechanics of plants, designing efficient biofuel conversion, and developing biomass-based products. Nevertheless, complete elucidation of the structure of cellulose and its interaction with matrix components has not been possible due to the complexity and heterogeneity of the cell wall and its variability from species to species. Ambiguity in the interpretation of structural characterization data obtained from different plant sources could sometimes be explained by a complimentary technique. For example, in the case of crystal parameters of native cellulose, the ambiguity in XRD results were resolved by NMR spectroscopy, which established the existence of two cellulose allomorphs, cellulose Iα and Iβ.

Ambiguities can also be seen among results quantifying certain properties measured through different techniques. For example, there is a large mismatch between estimates of the lateral dimension of cellulose crystallites obtained from XRD and from electron microscopy. Most often, this mismatch is attributed to artifacts induced by sample preparation for electron microscopy. Due to the structural complexity, a single technique cannot characterize a cellulose microfibril completely. Recent reports present cell wall characterization through combined application of complementary techniques like diffraction, scattering, spectroscopy, and microscopy. This combination of techniques is also applied for examining the interaction between cellulose and other cell wall polysaccharides by either studying cellulose microfibrils after sequential removal of other polysaccharides (top-down approach) or by studying the effect on cellulose after introducing additives to bacterial cellulose composites (bottom-up approach). Recent developments in SS-NMR have also enabled the study of interactions of cell wall components with each other and with water directly in native primary cell walls of plants.

The perpetual concern of introduction of artifacts during cell wall preparation has been reduced with the application of techniques like AFM and X-ray scattering that require minimal sample preparation. These approaches allow for the characterization of cell walls in near native states. The use of hybrid techniques like AFM-IR/Raman and advanced scattering techniques like RSoXS can provide chemical sensitivity along with high spatial resolution. In addition, use of advanced microscopic techniques like cryogenic electron tomography (cryo-ET) can create 3D reconstructions of nearly native cell walls and use of image analysis tools can quantify aspects of the microstructure.

Despite tremendous progress to date, many aspects of cellulose structure, and cellulose–cellulose and cellulose-matrix interactions are not well understood. The relation between the nanostructure of the cell wall and its macroscopic properties remains elusive. The current model of the primary cell wall suggests the existence of ‘biomechanical hotspots,’ which are sites of close contacts between cellulose microfibrils mediated by xyloglucans (Cosgrove, 2014). These are proposed as the control sites for wall extension. Nevertheless, many questions regarding the creation, destruction, location, and functioning mechanism of these structures are yet to be answered. Furthermore, recent work shows a linear correlation between the FWHM of cellulose (200) diffraction peaks and *d*-spacing for different sources (Huang et al., 2018). Thus, the *d*-spacing of cellulose is inversely proportional to the crystallite size. This inverse proportionality might be from enhanced thermal fluctuations and higher para-crystalline disorder in smaller crystals; yet, further work is warranted to ascertain the origin of this empirical relationship. We predict that the application of emerging approaches and multi-modal analyses (combination of multiple techniques) will generate new insights on the abovementioned topics and on other open questions regarding the regulation of cell wall growth and mechanics.

## Author Contributions

EWG, EDG, DY, and SR contributed to the conception and design of the review. All authors wrote the manuscript, contributed to manuscript revisions, and read and approved the submitted version.

## Conflict of Interest Statement

The authors declare that the research was conducted in the absence of any commercial or financial relationships that could be construed as a potential conflict of interest.
